# Functionalized Sulfur-Containing Heterocyclic Analogs Induce Sub-G1 Arrest and Apoptotic Cell Death of Laryngeal Carcinoma In Vitro

**DOI:** 10.3390/molecules28041856

**Published:** 2023-02-15

**Authors:** B. Haridevamuthu, Tamilvelan Manjunathan, Carlton Ranjith Wilson Alphonse, Rajendran Saravana Kumar, Sundaram Thanigaivel, Somasundaram Chandra Kishore, Vickram Sundaram, Pushparathinam Gopinath, Jesu Arockiaraj, Stefano Bellucci

**Affiliations:** 1 Department of Biotechnology, College of Science and Humanities, SRM Institute of Science and Technology, Kattankulathur, Chennai 603203, Tamil Nadu, India; 2 Department of Chemistry, College of Engineering and Technology, SRM Institute of Science and Technology, Kattankulathur, Chennai 603203, Tamil Nadu, India; 3 Molecular and Nanomedicine Research Unit, Centre for Nanoscience and Nanotechnology, Sathyabama Institute of Science and Technology, Chennai 600119, Tamil Nadu, India; 4 Chemistry Division, School of Advanced Sciences, VIT University, Chennai Campus, Chennai 600127, Tamil Nadu, India; 5 Department of Biomedical Engineering, Saveetha School of Engineering, Saveetha Institute of Medical and Technical Sciences, Chennai 602105, Tamil Nadu, India; 6 Department of Biotechnology, Saveetha School of Engineering, Saveetha Institute of Medical and Technical Sciences, Chennai 602105, Tamil Nadu, India; 7 INFN—Laboratori Nazionali di Frascati, 00044 Frascati, Italy

**Keywords:** apoptosis, benzo[b]thiophene, anticancer, laryngeal cancer, pharmacology

## Abstract

In this study, we speculate that the hydroxyl-containing benzo[b]thiophene analogs, 1-(3-hydroxybenzo[b]thiophen-2-yl) ethanone (BP) and 1-(3-hydroxybenzo[b]thiophen-2-yl) propan-1-one hydrate (EP), might possess antiproliferative activity against cancer cells. Hydroxyl-containing BP and EP show selectivity towards laryngeal cancer cells (HEp2), with IC_50_ values of 27.02 ± 1.23 and 35.26 ± 2.15 µM, respectively. The hydroxyl group present in the third position is responsible for the anticancer activity and is completely abrogated when the hydroxyl group is masked. BP and EP enhance the antioxidant enzyme activity and reduce the ROS production, which are correlated with the antiproliferative effect in HEp-2 cells. An increase in the *BAX/BCL-2* ratio occurs during the BP and EP treatment and activates the caspase cascade, resulting in apoptosis stimulation. It also arrests the cells in the Sub-G1 phase, indicating the induction of apoptosis. The molecular docking and simulation studies predicted a strong interaction between BP and the CYP1A2 protein, which could aid in combinational therapy by enhancing the bioavailability of the drugs. BP and EP possess an antioxidant property with low antiproliferative effects (~5.18 µg/mL and ~7.8 µg/mL) as a standalone drug, therefore, they can be combined with other drugs for effective chemotherapy that might trigger the effect of pro-oxidant drug on healthy cells.

## 1. Introduction

Cancer is the second major cause of death worldwide and kills roughly one in six people. Around 70% of cancer deaths occur in low- and middle-income countries, mostly due to the scarcity of affordable treatments. The advancements in technology and the knowledge of neoplastic diseases could help with discovering new drugs for reducing the death rate due to cancer [1]. Cancer is a multiphase process that involves the transformation of the pre-cancerous lesion into malignant tumors. Physical, chemical, and biological factors interact together with natural genetic causes to change healthy cells into cancerous ones [2]. Head and neck cancer (HNC) is one of the most common types, and it makes up 5% of all malignant tumor worldwide [3]. Each year, approximately 0.89 million patients are diagnosed, with 51% capitulating to the disease, and this is estimated to increase further [4,5]. HNC is a disparate tumor that affects the oral cavity, larynx, and pharynx, with over 90% of cases being squamous carcinomas known as head and neck squamous cell carcinoma (HNSCC). Laryngeal squamous cell carcinoma (LSCC) is a subcategory of HNSCC that is becoming extremely prevalent, with the second-highest mortality rate among them [6,7]. LSCC is frequent in patients with a smoking history and is evidenced by changes in their voice, breathing, and swallowing difficulties and the blockage of the bronchial vocalization, all of which impair the patient’s quality of life [8]. The tumor-targeting therapeutic approach showed promising results recently, reducing the deleterious effects on healthy cells, while enhancing bioavailability [9]. Cancer arises from the dysregulation of the multicellular mechanisms required for proper cell proliferation such as apoptosis. In addition, most of the commonly used drugs do not have anticancer selectivity, which causes extreme adverse effects on normal tissues. Moreover, most patients will eventually develop drug resistance, leading to therapy failure. Drug efflux, altered drug metabolism, and reduced apoptosis are the proposed attributes of drug resistance in cancer [10]. The main goal of the modern cancer drug development pipeline is to identify and deliberately target the drug regulatory metabolism involved in cancer cell alterations [11].

Recently, the cost and time it takes to develop new drugs have increased dramatically, which means that if drug resistance develops, patients with severe illnesses may end up dying before the drugs are made available [12]. Drug repositioning or remodeling strategies are adapted to overcome this bottleneck in the cancer research community [13]. On the other hand, thiophene derivatives, especially naturally occurring heterocyclic benzo[b]thiophene, has a wide application in medicinal chemistry that draws the attention of researchers [14]. The aromaticity of benzo[b]thiophene makes it a stable molecule and its heterocyclic reactive sites could be functionalized by a modification that acts as an excellent scaffold to be remodeled as a cancer drug [14]. The Food and Drug Administration (FDA) of the United States of America approved Raloxifene hydrochloride with a benzo[b]thiophene scaffold [14,15]. Sertaconazole, Zileuton, and Benocyclidine are some other commercial drugs that have benzo[b]thiophene in their core [16]. Recent studies also indicate that acrylonitrile-containing benzothiophene [17] and amino benzothiophene analogs [18] exhibit potent anticancer properties via disrupting polymerization and cell cycle processes. Benzothiophene-2-hydroxamic acid was found to be an inhibitor of histone deacetylase and exhibits inhibition against colon cancer cells (HCT116) [19]. Researchers also found that cyanide and imidazolinyl-substituted benzothiophene analogs showed specificity toward the inhibition of HeLa cells [20]. Here, we have followed a one-pot strategy starting with 2,2 dithio dibenzoylchloride to synthesize two hydroxyl-containing analogues, 1-(3-hydroxybenzo[b]thiophen-2-yl) ethanone (BP) and 1-(3-hydroxybenzo[b]thiophen-2-yl) propan-1-one hydrate (EP), and further mask the hydroxyl group with acetyl chloride and methyl iodide to obtain 2-acetylbenzo[b]thiophene-3-yl acetate (BN) and 1-(3-methoxybenzo[b]thiophene-2-yl) propan-1-one (EN) [21,22]. Hydroxyl-containing analogs BP and EP were predicted to be CYP1A2 inhibitors in our previous report [22]. Therefore, we speculate that the synthesized benzo[b]thiophene analogs with a hydroxyl moiety might possess an anticancer effect. To test our hypothesis, we initially screened the anticancer activity of both hydroxyl-containing (as a positive group) and hydroxyl-masked (as a negative group) benzo[b]thiophene analogs in four human cancer cell lines, namely, human laryngeal carcinoma cells (HEp2), human breast cancer cells (MCF-7), human gastric adenocarcinoma cells (AGS), and human osteoblast tumor cells (MG63). We found that analogs with a hydroxyl moiety (BP and EP) were selective against HEp2, so we have expanded our further study on BP and EP with IC_20_ and IC_50_ concentrations to unveil the molecular mechanism of HEp2 cell proliferation inhibitory action. To gain insight into the molecular dynamic behavior of the analogs at the active site of CYP1A2, we performed an in silico molecular dynamics (MD) simulation analysis.

## 2. Results and Discussion

### 2.1. Cytotoxic Effect of the Benzo[b]thiophene Analogs

The potential inhibitory activity of the analogs BP, BN, EP, and EN were assessed against HEp2, MCF7, AGS, and MG63 cell lines using an MTT reagent, and the 50% inhibiting concentration (IC_50_) of the analogs were also calculated (Table 1). 

**Figure 1:**
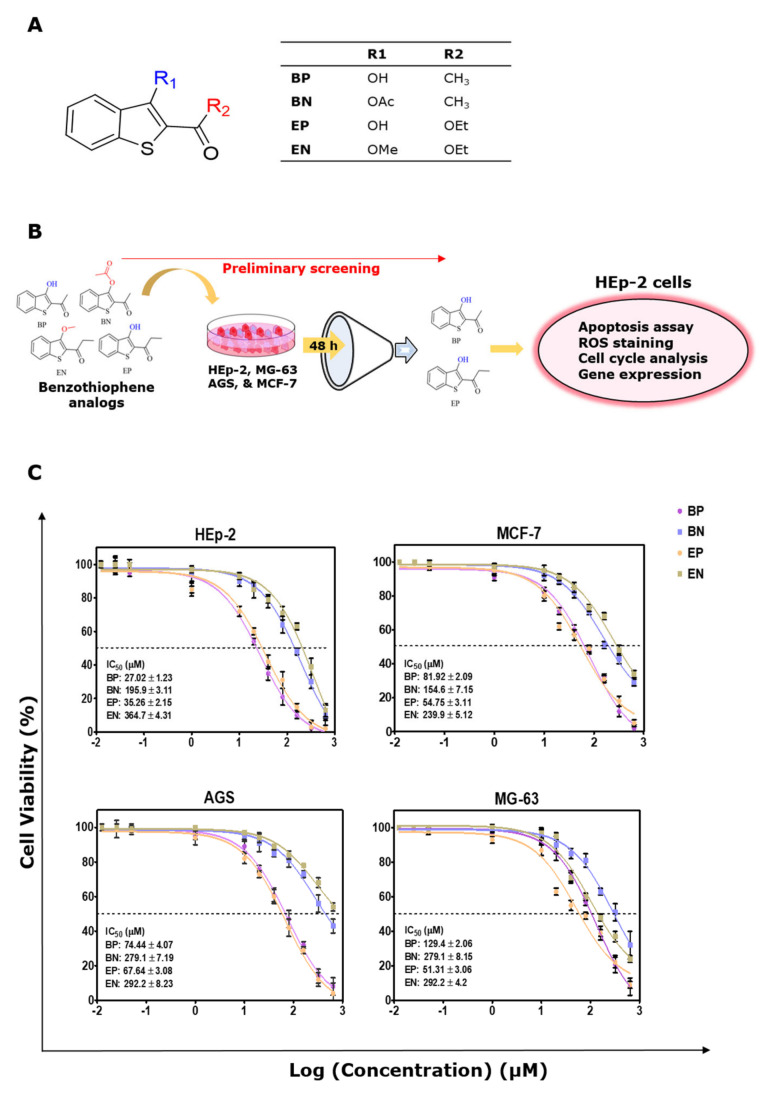
(**A**) Structure of the benzo[b]thiophene analogs. (**B**) Experimental outline of this study. (**C**) Logarithmic dose–response curves of Benzo[b]thiophene analog treatment in human laryngeal carcinoma (HEp−2), breast adenocarcinoma (MCF7), gastric adenocarcinoma (AGS), and osteosarcoma (MG63) cell lines. Cells were cultured in 96-well plates and treated with benzo[b]thiophene analogs for 48 h, and the viability was measured by MTT assay. IC_50_ values were represented as mean ± SD of three independent experiments.

Structures BP, BN, EP, and EN are represented in Figure 1A, along with the experimental design in Figure 1B. A dose-dependent decrease in cell proliferation was observed (Figure 1C). The IC_50_ values of analogs BP and EP against the HEp2 cells were about 27.02 ± 1.23 µM (~5.18 µg/mL) and 35.26 ± 2.15 µM (~7.8 µg/mL), which are relatively (*p* < 0.01) lower than those of BN (195.9 ± 3.11 µM; ~45.84 µg/mL) and EN (364.7 ± 4.31 µM; ~86.45 µg/mL). The IC_50_ of all the analogs against the MCF7, AGS, and MG63 cells was found to be greater than 50 µM, which is generally considered to be less effective. Analogs BP and EP are more effective at inhibiting HEp2 cell proliferation than other cancer cells used in this study were. Analogs BN and EN were not effective against any of the cancer cells used. The cytotoxicity of these analogs was also evaluated in a non-tumorigenic cell line (Vero), and the selectivity index (SI) values of the analogs were calculated (Table 1). The SI value indicates the safety of the analogs screened for antiproliferative activity. Analogs BN and EN showed an SI value of lower than 1, indicating that the analogs BN and EN exhibit a more cytotoxic effect on non-tumorigenic cells than they do on cancer cells. A high SI value denotes the safety of the anticancer drugs. BP has the higher SI value of 5.13 compared (*p* < 0.01) with that of EP at 2.94. 

**Figure 2:**
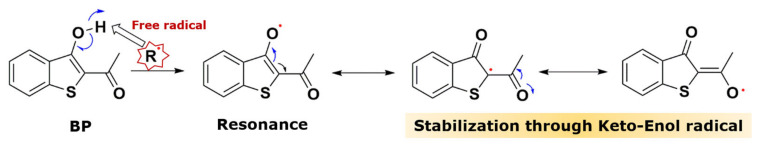
Redox mechanism of analog BP during free radical attack and stabilization through keto-enol radical resonance was denoted using half arrow.

In comparing the bioactivity of BP and EP, BP showed better activity in terms of controlling the proliferation of cancer cells as well as therapeutic safety than EP did. Hence, we anticipate that the possession of the ester group in EP reduces the bioactivity more compared to that of BP. There are many reports showing that the presence of ester group in compounds induces anticancer activity [23]. Analogs BP and EP showed a seven-fold and a nine-fold increase in activity compared to those of hydroxyl-masked BN and EN, respectively. This denotes that the presence of the hydroxyl group in the third carbon may be responsible for the growth inhibition of the HEp2 cells. The substitution of the hydroxyl group with other group abrogated its anticancer, and also, antioxidant activities. Previous studies have also confirmed that the substitution of the hydroxyl group of triptolide with an acetyl group nullified its anticancer activity [24]. It has already been stated that the presence of a hydroxyl group in the third or fifth positions of a structural element is responsible for free radical scavenging [25] and is also associated with anticancer potential [26]. While examining the antioxidant potential in our previous study [22], BP, which possesses the ketone group in the 2nd position, shows better activity compared to that of EP, which possesses the ester group in the 2nd position; the better activity profile of BP is due to the resonance stability of the keto-enol form, where the ketone group has lower pKa value compared to that of the ester group, since this is more acetic than EP is [22]. BP has better stability in keto-enol tautomerism and shows better activity compared to that of EP (Figure 2) [22,27]. Various studies have shown that a drug scaffold bearing keto-enol functionality possesses anticancer effects [28,29]. Since there are no possibilities for molecules BP and EP to generate superoxide radicles, unlike ortho-quinone or catechol moieties [30], we strongly believe that the analogs BP and EP exhibit their function as an antioxidant instead of a pro-oxidant in inducing cellular apoptotic mechanisms to inhibit the proliferation of HEp2 cells.

**Figure 3:**
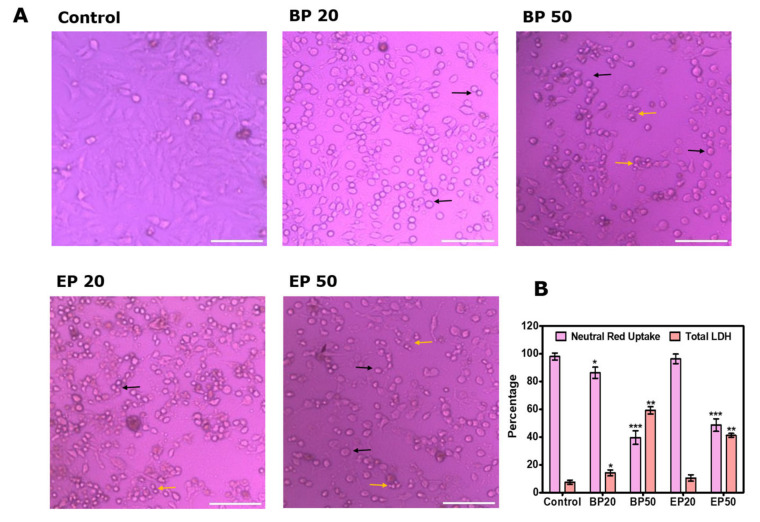
Cytotoxic effect of BP and EP on Hep-2 cells. (**A**) Morphological changes of the HEp2 cells after treatments with BP and EP at IC_20_ and IC_50_ concentrations. The cells were analyzed using a brightfield inverted microscope at 100× magnification. Morphological changes such as cell rounding (indicated by a short black arrow) and cell shrinkage (indicated by a short yellow arrow) were observed with the BP and EP treatments. (**B**) Neutral red uptake (NRU) and total LDH release of the HEp2 cancer cells. Reduction of NRU and increase in LDH indicate the cytotoxic ability of analogs BP and EP. Data are expressed as mean ± SD of three independent experiments. * *p* < 0.05, ** *p* < 0.01, *** *p* < 0.001 significance difference between the control and treatment. Scale: 50 µm.

The antiproliferative activity of the analogs BP and EP are related to the apoptotic pathway, as observed under the brightfield microscope, which showed the morphological changes in the cells after the treatment (Figure 3A). The adherence of the cells was found to be modulated better when they were treated with analogs BP and EP at IC_50_ compared with that of the untreated control group. Cell rounding was observed even at the IC_20_ concentration, and shrinkage was noticed in the cells at the IC_50_ concentration (Figure 3A), which shows that the analogs could activate the apoptotic mechanism at a lower concentration. In addition, cells that no longer adhered to the substrate detached from the plates, as seen previously with synthetic 2-Phenylnaphthalenes [31]. We also evaluated the cytotoxicity of analogs BP and EP by performing a neutral red (NR) uptake assay to confirm lysosomal integrity (Figure 3B). NR stains the intact lysosomes of viable cells. The percent of uptake of the NR stain is directly related to the viability of the cells. We found a decrease in NR uptake, showing that analogs BP and EP have a cytotoxic effect against HEp2 cells, which is a similar result as that which was found during the MTT. BP and EP at IC_50_ more significantly (*p* < 0.01) decreased the NR uptake by 39.65 ± 4.9% and 48.65 ± 4.5% compared to that of the control. It proves that the lysosomal membrane permeability was disrupted by the analogs BP and EP. LDH is released by damaged cells and could act as a biomarker for cellular damage. The percentage of total LDH released by the treated HEp2 cells with BP and EP was significantly (*p* < 0.01) higher than that of the control, showing the damage caused by BP and EP to the HEp2 cancer cells (Figure 3B). The BP at IC_50_ showed about 59.25 ± 2.6% of LDH release, which is 7.9 fold increase from that of control. The concentration of EP at IC_50_ was about 41.24 ± 1.4%, which is 5.5 fold increase compared to that of the control.

### 2.2. Alteration in ROS Status by BP and EP Analogs

**Figure 4:**
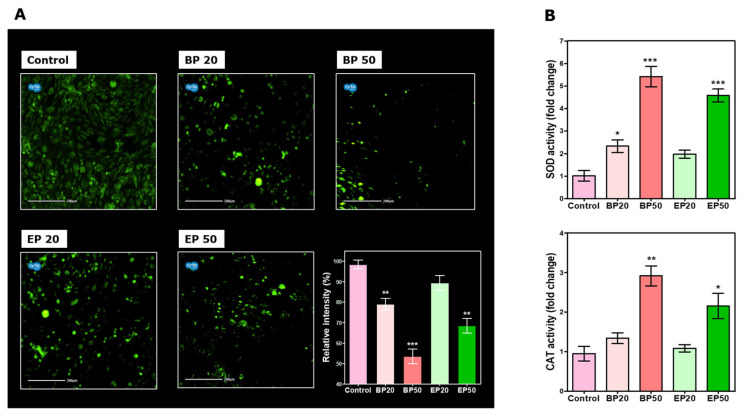
Redox imbalance caused by analogs BP and EP in Hep-2 cells. (**A**) Intracellular ROS production stained by DCF-DA captured by CytoSMART® Lux3 FL fluorescence microscope. (**B**) SOD and CAT enzyme activities. A decrease in ROS causes an imbalance in the redox status of the cancer cells, causing the induction of apoptosis. Data are expressed as mean ± SD of three independent experiments. * *p* < 0.05, ** *p* < 0.01, *** *p* < 0.001 significance difference between the control and treatment. Scale: 200 µm.

The hydroxyl-containing BP and EP analogs are already known for their antioxidant activity. Antioxidant molecules prevent the free radical damage that is associated with cancer development and progression. Scavenging ROS in cancer cells decreases their ability to balance oxidative insults and might result in the activation of apoptosis [32]. We used an ROS-specific fluorescent probe, DCF-DA, to measure the ROS in the HEp-2 cells (Figure 4A). We observed a significant decrease in the relative fluorescence intensity at IC_50_ of the BP (*p* < 0.001) and EP (*p* < 0.01) treatments. BP50 reduced about 45 ± 5.65% and EP50 reduced about 30 ± 1.4% of the ROS compared to that of the control. Similar results were reported with the anticancer activity of curcumin, which reduces the ROS production and induces mitochondrial-mediated cell death [33]. This is evident by the increase in SOD and CAT activity (Figure 4B). BP50 increased the SOD and CAT activities of HEp-2 cells by 5.42 ± 0.45 fold (*p* < 0.001) and 2.92 ± 0.25 fold (*p* < 0.01), respectively, whereas EP50 increased the SOD and CAT activities of HEp-2 cells by 4.58 ± 0.28-fold (*p* < 0.001) and 2.155 ± 0.31-fold (*p* < 0.05), respectively, compared to those of the control. Free radicals generated by the healthy cellular metabolism are detoxified by SOD- and CAT-maintaining cellular homeostasis, while a higher level of ROS results in the activation of apoptosis or cell death [34]. The results obtained here are corroborated by Huang et al. (2015), who found that a natural tropolone compound named hinokitiol exerts anticancer activity through enhancing SOD and CAT enzyme activities and reducing the hydroxyl radicals [34]. Cancer cells have higher levels of oxidative stress as compared to that of healthy cells, allowing them to activate several signaling pathways and transcription factors that are able to promote tumor growth and malignant progression [35]. Analogs BP and EP might cause redox homeostasis imbalance in the HEp-2 cells, resulting in halted cell progression due to the loss of several ROS-mediated signals, thereby inducing apoptosis [36]. A previous study on curcumin also stated similar results, with reduced ROS production in cancer cells, which lead to cell death [34]. 

### 2.3. Activation of Cellular Apoptosis by Hydroxyl-Containing BP and EP Analogs

**Figure 5:**
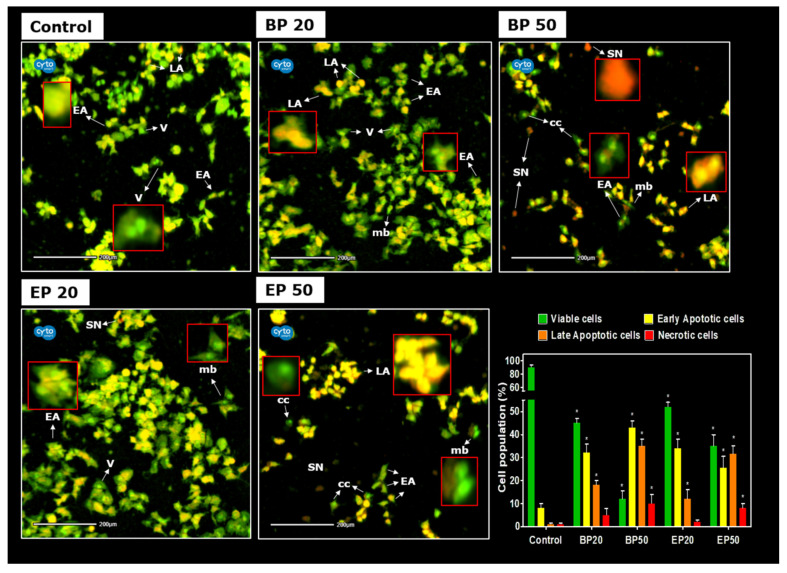
Fluorescent images of AO/PI dual-stained HEp2 cells after treatments with BP and EP at IC_20_ and IC_50_ concentrations for 48 h captured by CytoSMART® Lux3 FL fluorescence microscope. Green nucleus represents viable cells, early apoptotic cells show yellow color, and late apoptotic cells display orange color. Intense red color cells indicate the secondary necrotic condition. The bar graph represents the percentage of cell population in apoptotic phases. The results are expressed as mean ± SD of triplicate experiments. * *p* < 0.05 significance difference between the control and treatment. (V, viable cells; EA, early apoptosis; LA, late apoptosis; SN, secondary necrosis; mb, membrane blebbing; cc, chromatin condensation). Scale: 200 µm.

Cell death via the apoptotic pathway can be distinguished by the change in morphological characteristics. To evaluate the ultramorphological changes, the cells were stained with nuclear dual stains AO/PI (Figure 5). AO is cell permeable and is taken up by both viable and non-viable cells, emitting a green-colored fluorescence. PI is a cell impermeable dye that could be taken up by the membrane-compromised cell, stains dead cells, and emits red fluorescence. Healthy viable cells (V) contain a green nucleus, as shown in the untreated control in Figure 5. Early apoptotic (EA) cells had a yellow color due to intense green fluorescence and also displayed condensed chromatin (cc) in EP50 (Figure 5), as well as membrane blebbing (mb) in EP20 (Figure 5). Late apoptotic (LA) cells emit both green and red fluorescence, thus displaying orange-colored fragmented or condensed chromatin, as shown the BP- and EP-treated group in Figure 5. Intensely red-colored cells indicate the secondary necrotic condition in BP50 (Figure 5). As we can see, the apoptotic conditions were enhanced when the cells were treated with BP and EP, which is indicated by the presence of yellow and red dots in the cells. The percentage of healthy viable cells was drastically decreased (*p* < 0.01) by the BP50 (12 ± 3.5%) and EP50 (35 ± 5%) treatments compared to that of the control (90 ± 4%). The BP50 and EP50 treatments contained 43 ± 3% and 25.5 ± 5% of the EA cells, 35. ± 3% and 31.5 ± 3.5% of the LA cells, and 10 ± 4% and 8 ± 2% of the necrotic cells. These changes are statistically significant (*p* < 0.01) when they are compared to the control, which has about 8 ± 2% EA cells, 1 ± 0.5% LA cells, and 1 ± 0.5% necrotic cells. The treatments with BP20 and EP20 for 48 h shifts these values to 32 ± 4% and 34 ± 4% EA cells, 18 ± 2% and 12 ± 4% LA cells, 5 ± 3% and 2 ± 1% necrotic cells, respectively. This shows that the analogs BP and EP, even at IC_20_, could induce apoptotic conditions, as observed at IC_50_. The structure activity relationship conducted by Chang et al. (2015) reported that the presence of a hydroxyl group in the third position of synthetic 2-Phenylnaphthalenes increases the rate of apoptosis in breast cancer cells, but the presence of the hydroxyl group in the third position decreased the cytotoxicity [31]. With our obtained result, we can also state that the presence of a hydroxyl group in the third position may be responsible for the low cytotoxic effect of BP and EP to cancer cells. The structural modification of these analogs might potentially increase the antiproliferative effect. 

### 2.4. Sub-G1 Cell Cycle Arrest by Hydroxyl-Containing BP and EP Analogs

**Figure 6:**
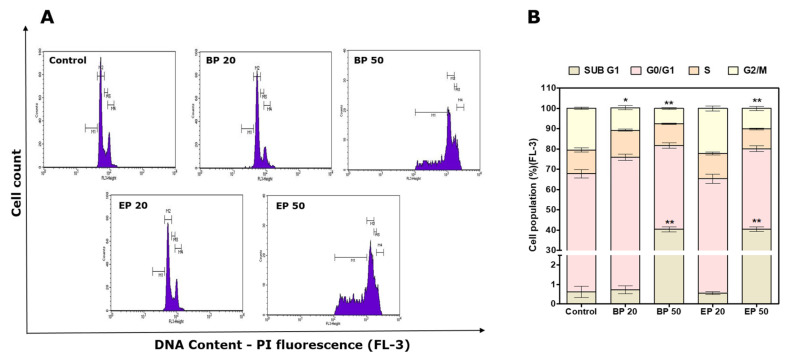
Cell cycle analysis by DNA content estimation with flow cytometer in HEp2 cells after treatments with BP and EP at IC_20_ and IC_50_ concentrations for 48 h. (**A**) Histograms show the cell distribution in the different phases of the cell cycle. (**B**) Bar graph indicating the percentage of cells in Sub-G1, G0/G1, S, and G2/M phases, respectively. The results are expressed as mean ± SD of triplicate experiments. * *p* < 0.05, ** *p* < 0.01 significance difference between the control and treatment.

The cell cycle of the HEp2 cells after the treatments was analyzed to further study the apoptotic pathway triggered by the analogs BP and EP (Figure 6). Cancer-specific drugs could cause cell death by blocking the checkpoints in the cell cycle, such as the Sub-G1 phase, G0/G1 phase, S phase, and G2/M phase [37]. Arrest in the Sub-G1 phase indicates the induction of apoptosis [38]. A histogram obtained from the flow cytometer showed an arrest of 40.4 ± 1.3% when the cells were treated with BP50 and 40.45 ± 1.2% when the cells were treated with EP50 in the Sub-G1 phase, which was significantly (*p* < 0.01) higher than that of the control (0.62 ± 0.3%) (Figure 5B and Figure 6A). The arrest in the Sub-G1 phase indicates an apoptotic pathway, proposing that the analogs BP and EP may act by a specific cell cycle mechanism inducing apoptosis in HEp2 cells. This arrest in the Sub-G1 phase leads to a decreased (*p* < 0.01) cell population in the G0/G1 phase, with 41.31 ± 1.4% of the cells in the BP50 group, 39.67 ± 1.4% of the cells in the EP50 group, and 67.25 ± 2% of the cells in the control group. Previously, Sun et al., (2010) reported that suberoylanilide hydroxamic acid with hydroxyl moiety induces Sub-G1 cell arrest in colon cancer cells [39]. This correlates with our current findings that the presence of the hydroxyl group may be responsible for inducing apoptosis in HEp-2 cells.

### 2.5. Increase in BAX/BCL-2 Transcript and Caspase Gene Expression by Hydroxyl-Containing BP and EP Analogs

**Figure 7:**
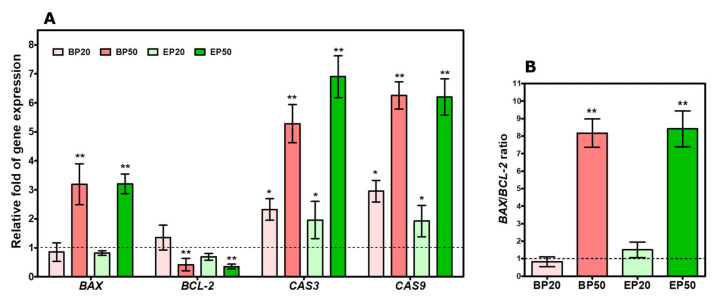
Effect of BP and EP on apoptotic gene expression of HEp2 cell line after 48 h treatment. (**A**) Expression profile of *BAX, BCL-2, CASPASE-3,* and *CASPASE-9* mRNA (normalized with *GAPDH*). (**B**) *BAX/BCL2* ratio increased with BP50 and EP50 treatment. The thin horizontal line represents the normalization of the control. The results are expressed as mean ± SD of triplicate experiments. * *p* < 0.05, ** *p* < 0.01 significance difference between the control and treatment.

We also studied the expression of the pro-apoptotic gene, as well as antiapoptotic genes, to unearth the mechanism of apoptosis activation by BP and EP (Figure 7A). To increase caspase activation, the *BAX/BCL-2* ratio is required to initiate apoptosis. BAX is a pro-apoptotic mediating factor for p53-related apoptosis, and *BCL-2* is a pro-survival gene necessary for the survival of the cells [40]. A decrease in the expression of *BCL-2* and the activation of *BAX* results in apoptotic conditions [41]. As a result, significant increases in apoptotic-related genes *CASP3* (2.32 ± 0.37- and 1.95 ± 0.64-fold, respectively) and *CASP9* (2.95 ± 0.3- and 1.92 ± 0.5-fold, respectively) were found in the BP20 and EP20 treatments, but there is no significant induction of *BAX* and *BCL-2* gene expression (Figure 7A). Increasing the concentration of BP and EP increased the induction of *BAX* expression by 3.19 ± 0.71-fold in BP50 and 3.2 ± 0.34-fold in EP50 and decreased *BCL-2* expression by 0.42 ± 0.21-fold in BP50 and 0.35 ± 0.08-fold in EP50, respectively. *BAX* activation could provoke the mitochondrial membrane permeabilization and activate the caspase cascade [42]. The caspase cascade plays a crucial role in apoptosis regulation [43]. Here, the *BAX/BCL-2* ratio was increased by 8.16 ± 0.8-fold in BP50 and 8.41 ± 1.02-fold in the EP50 treatment (Figure 7B). This leads to an increase in caspase activation, 5.25 ± 0.6-fold of *CASP3* and 6.25 ± 0.47-fold of *CASP9* in BP50 and 6.9 ± 0.72-fold of *CASP3* and 6.2 ± 0.63-fold of *CASP9* in EP50. The increased *BAX/BCL-2* ratio in this study was related with the anticancer effects of synthetic 2-Phenylnaphthalenes [31] and 7α-hydroxy-β-sitosterol [44]. Additionally, several natural compounds such as epigallocatechin-3-gallate and resveratrol influence the *BAX/BCL-2* ratio and exerts its anticancer effects [44]. In reference to previous reports, the results in this study also suggest that BP and EP induces apoptosis in HEp-2 cells through the dysregulation of the *BAX/BCL-2* ratio, possibly inducing caspase-8 and 9. 

### 2.6. BP Possibly Interacts with Human CYP1A2 in MD Simulation

**Figure 8:**
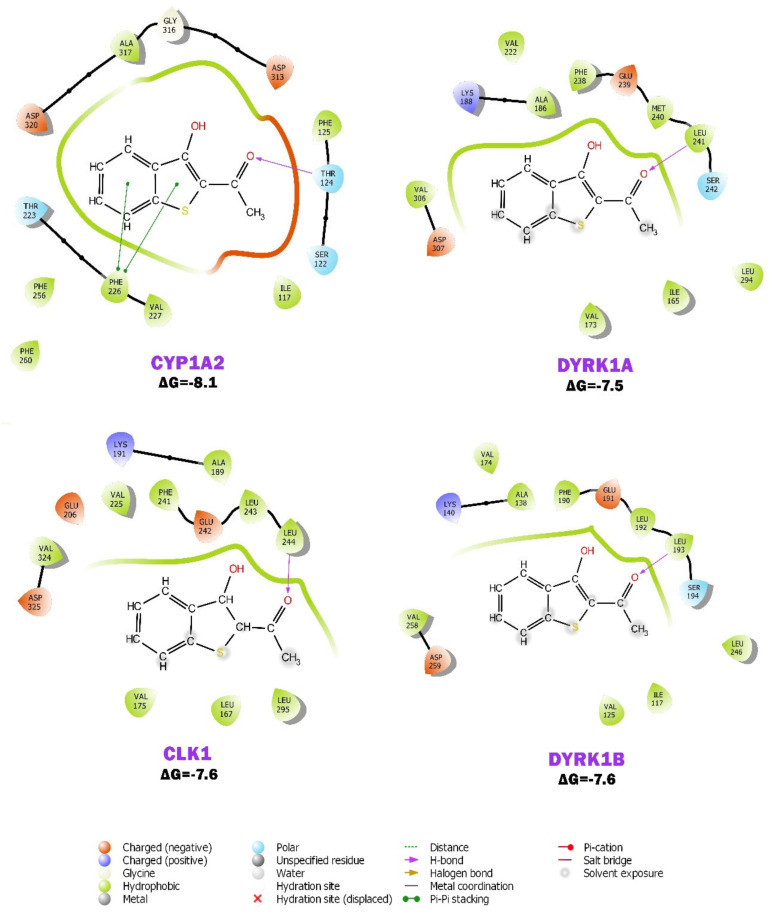
The ligand interaction diagram of compound BP with human CYP1A2, DYRK1A, CLK1, and DYRK1B. Binding energy values (ΔG, kcal/mol) of BP with CYP1A2, DYRK1A, CLK1, and DYRK1B were predicted to be −8.1, −7.5, −7.6, and −7.6, respectively. The lowest energy was observed with CYP1A2.

Molecular docking and dynamic simulation studies were performed between analog BP and the predicted target proteins, human CYP1A2, DYRK1A, DYRK1B, and CLK1. CYP1 family is involved in various xenobiotic metabolism, including metabolizing tobacco procarcinogen into carcinogenic products [45]. It metabolizes anticancer agents, such as docetaxel, paclitaxel, and cisplatin, and has been linked to drug resistance [46]. More evidence showed that CYP1s are overexpressed in various cancers [47]. CYP1A2 is induced by multiple factors, including smoking, which leads to the loss of therapeutic effects of substrate drugs, and it contributes markedly to the enhancement of the risk of HNC [48]. This study predicts that BP significantly interacts with CYP1A2 based on the interacting residue, favorable binding orientation, significant interactions, interaction energy, binding affinity, and involved forces (Figure 8). The binding energy values (ΔG, kcal/mol) of BP with CYP1A2, DYRK1A, CLK1, and DYRK1B were observed to be −8.1, −7.5, −7.6, and −7.6, respectively. The lowest energy value was predicted with CYP1A2. 

**Figure 9:**
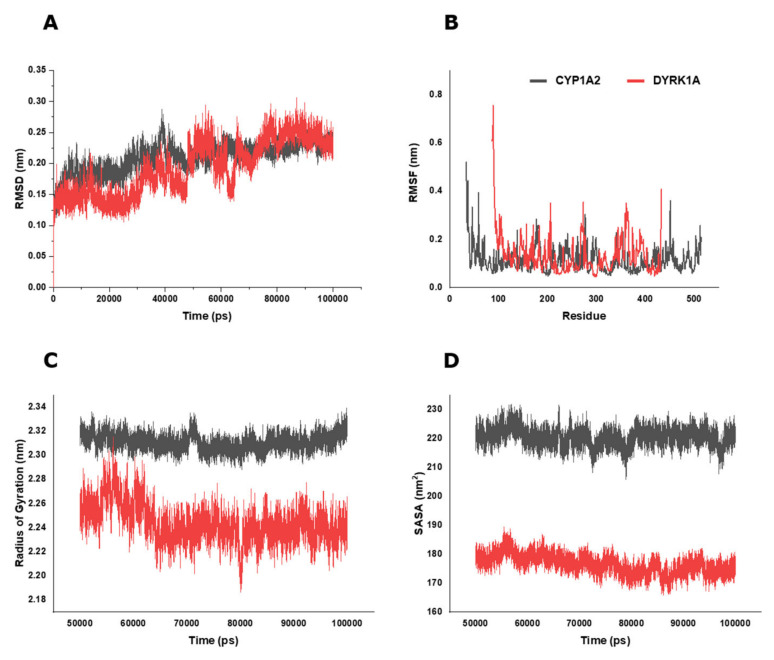
Molecular simulation dynamics of CYP1A2 (Grey) and DYRK1A (Red) protein and compound BP. (**A**) Plot of time vs. RMSD of CYP1A2 and DYRK1A protein relative to the starting complexes during 10000 ps MD test for compound BP. (**B**) RMSF plot of CYP1A2 (Grey) and DYRK1A (Red) protein during 10,000 ps MD represents local changes along the protein chain. (**C**) Radius of gyration (Rg) value and (**D**) solvent-accessible surface area (SASA) plot value during the computed 10,000 ps of time for all the systems.

Using the docking analyses, MD simulations were performed for BP and CYP1A2 and DYRK1A, and their dynamic behavior was analyzed with respect to time (Figure 9). RMSD (root-mean-square deviation) analysis is the fluctuation of the protein from its initial position in the simulation to its final conformation after a given time to determine the protein’s stability (Figure 9A). The CYP1A2-BP complex reached equilibrium and remained stable throughout the simulation period, while the DYRK1A-BP complex was not stable, fluctuating throughout the run. RMSF (root-mean-square fluctuation) data were used to analyze the structural flexibilities of the protein brought by BP during the 100,000 ps simulation (Figure 9B). No significant changes in the protein backbone stability were found with the CYP1A2-BP complex. The DYRK1A-BP complex showed a deviation of residue over time. Analog BP was investigated to study the radius of the gyration effect of the ligand–protein complex and was found to be in the range of 2.28–2.33 nm for CYP1A2 and 2.18–2.31 nm for DYRK1A during the simulation (Figure 9C). Solvent-accessible surface area (SASA) analysis measures the region of the protein that is accessible to the solvent molecules (Figure 9D). The SASA value of CYP1A2 was analyzed to be 205.72–231.82 nm^2^, which is significantly higher than that of the DYRK1A-BP complex (165.69–189.44 nm^2^). The energetic components in the MD simulation showed a better interaction of BP with CYP1A2 than with DYRK1A (Figure S14 from the Supplementary Materials). In silico studies predicted that the analog BP might be a better inhibitor of the CYP1A2 enzyme than DYRK1A is. Studies have found that an antioxidant molecule named Pinocembrin also have potential antiproliferation activity against breast cancer cells [49], and also was found to be a CYP1A2 inhibitor [50]. From the results obtained in this study, we can strongly suggest that BP and EP action resembles the mechanism of action of Pinocembrin by having an antioxidant potential, and they possibly could bind to CYP1A2, thus exerting anticancer activity. Therefore, the design, synthesis, and development of a prodrug is possible with the benzo[b]thiophene scaffold, which actually might introduce a novel approach to cancer therapy [51].

## 3. Materials and Methods

### 3.1. Cell Culture Maintenance

Human laryngeal carcinoma cells (HEp2), human breast cancer cells (MCF-7), human gastric adenocarcinoma cells (AGS), human osteoblast tumor cells (MG63), and monkey kidney epithelial cells (Vero) were obtained from National Centre for Cell Science (NCCS), Pune, India. All the cells were grown as a monolayer culture in a passage medium containing high-glucose Dulbecco’s Modified Essential Medium (DMEM) (Gibco, Oxford, UK) with 4.5 g/L glucose, 10% Fetal Bovine Serum (FBS) (Gibco), and 0.1% antibiotic–antimycotic solution (Sigma, St. Louis, MO, USA), which was kept in a humidified incubator at 37 °C with 5% CO_2_. The fresh medium was changed every 48 h, and all the cells were routinely passaged using Trypsin-EDTA (Sigma) when 70% confluence was reached. 

### 3.2. Assessment of Cell Growth Inhibition after Benzothiophene Analogs Treatment

MTT (3-(4,5-Dimethylthiazol-2-yl)-2,5-Diphenyltetrazolium Bromide) reagent (Sigma, USA) was used to evaluate the antiproliferative activity of hydroxyl-containing positive analogs BP and EP and hydroxyl-masked negative analogs BN and EN on the four cancer cell lines [52]. HEp2, MCF-7, AGS, and MG63 cells were seeded at around 5 × 10^3^ cells/well in distinguished 96-well plates and replenished with fresh complete DMEM to reach confluence. Then, the cells were exposed to the analogs at different concentrations for 48 h in serum-free media. Twenty µL of MTT (5 mg/mL) was added to the treated wells and incubated in dark for 4 h at room temperature (RT) and subjected to mild shaking. The MTT-containing medium was removed carefully, and DMSO was used to dissolve the formazan crystal formed inside the cell due to a mitochondrial reaction. The absorbance was read at 650 nm using a Multiskan Go ELSIA reader (Thermofisher Scientific, Vantaa, Finland). The logarithmic dose–response curves for each cell line were fitted, and the concentration of the analogs required to inhibit the cell proliferation at 20% (IC_20_) and 50% (IC_50_) values were calculated using the best fit value obtained by the non-linear regression using GraphPad Prism software (Ver 5.03, La Jolla, CA, USA).

### 3.3. Neutral Red Uptake after Treatment with BP and EP

BP and EP analogs were chosen for further studies based on the selectivity index towards HEp2 cells. HEp2 cells were seeded at around 5 × 10^3^ cells/well in 96-well plates and incubated at 37 °C with 5% CO_2_ to reach 70% confluence, then exposed to the IC_20_ and IC_50_ of BP and EP for 48 h in serum-free media. After exposure, the cells were washed thrice with PBS and incubated in a 0.3% neutral red (NR) solution for 3 h. The cells were washed again with PBS, destained with a solution containing 50% ethanol and 1% glacial acetic acid, and subject to mild shaking for 30 min to remove the excessive NR solution. Finally, the absorbance was read at 540 nm using a Multiskan Go ELSIA reader (Thermofisher Scientific), and the cell viability was calculated as previously reported [52].

### 3.4. Total Lactate Dehydrogenase (LDH) after Treatment with BP and EP 

The cell membrane permeability after the analog treatment was evaluated by the amount of LDH released in the media [53]. Fifty µL of media from the treated cells was transferred to a new 96-well plate, and fifty µL of 5% Triton X-100 (HiMedia, Mumbai, India)was added to the original 96-well plate to calculate the total LDH. One hundred µL of 4.6 mM pyruvic acid (prepared in 0.1 M potassium phosphate buffer, pH 7.5) was added to both plates, followed by 100 µL of 0.5 mg/mL-reduced NADH. The change in absorbance value was read for 2 min at 340 nm in a kinetic setting using a Multiskan Go ELISA reader (Thermofisher Scientific) and used for calculating LDH release as per the previous report [52].

### 3.5. Visual Morphological Changes after Treatment with BP and EP 

Visual morphological changes in the HEp-2 cells that occurred during the analog exposure were examined under a brightfield microscope. Briefly, the cells were seeded in a 6-well plate and allowed to grow overnight. Then, the cells were exposed to IC_20_ and IC_50_ of BP and EP for 48 h and washed with media. The brightfield images were captured using a USB digital camera (MDCE-5C, USB 2.0) mounted on an inverted microscope at 40× magnification using ScopeImage 9.0 software.

### 3.6. Intracellular ROS Measurement after Treatment with BP and EP

Intracellular ROS generated in the HEp2 cells were visualized using a 2′, 7′-dichlorodihydrofluorescein diacetate (DCF-DA) fluorescent probe [54]. Briefly, the HEp-2 cells were seeded at 1 × 10^5^ cells/ well in a 6-well plate and incubated for 24 h or until they reached confluence, after which, they were exposed to IC_20_ and IC_50_ of BP and EP for 48 h in serum-free media. The cells were washed thoroughly and stained with 10 µM of DCF-DA and incubated in dark for 40 min at RT. Then, the cells were washed extensively with PBS and visualized under a CytoSMART^®^ Lux3 FL fluorescence microscope (CytoSMART^®^ Technologies, Eindhoven, The Netherlands) at 20× digital zoom, and fluorescence signals were evaluated using ImageJ software. 

### 3.7. SOD and CAT Activity of HEp-2 Cells

SOD enzyme activity was determined by a nitroblue tetrazolium (NBT) reduction assay [55]. Briefly, 1 × 10^4^ cells/well were seeded in 96-well plates and incubated for 24 h or until they reached confluence, after which they were exposed to IC_20_ and IC_50_ of BP and EP for 48 h. NBT was added to each well and incubated for 3 h at RT. The formazan crystals formed were solubilized in DMSO, after that, the absorbance at 550 nm was measured, and the results were plotted relative to the SOD activity compared to that of the untreated HEp-2 cells (control). CAT activity was estimated based on hydrogen peroxide (H_2_O_2_) decomposition, as previously described [56]. The cells after the treatments were lysed in Tris-HCl buffer (100 mM, pH 7.4) using an ultrasonic homogenizer. The cell lysate was mixed with 50 mM of phosphate buffer and 30 mM of H_2_O_2_. The change in the absorbance value was recorded at 240 nm, and CAT activity was calculated as previously described [22].

### 3.8. Ultramorphological Assessment of Apoptosis by Live/Dead Double Staining after Treatment with BP and EP

The apoptosis changes in the treatment were determined by staining the cells with acridine orange/propidium iodide (AO/PI) fluorescence dyes. Briefly, the HEp-2 cells were seeded at 1 × 10^5^ cells/ well in a 6-well plate and incubated for 24 h or until they reached confluence. Then, the cells were exposed to IC20, IC50 of BP, and EP for 48 h. After the exposure period, the cells were stained with 25 µL of AO/PI dye mixture (100 µg/mL). The cells were washed extensively with PBS and visualized under a CytoSMART^®^ Lux3 FL fluorescence microscope (CytoSMART^®^ Technologies, Eindhoven, The Netherlands) at 20× digital zoom, and the fluorescence positive cells were counted using the CytoSMART Object Count algorithm.

### 3.9. Change in Cell Cycle Analysis after Treatment with BP and EP

Changes in the cell cycle after the treatment were analyzed using a flow cytometer. Briefly, 1 × 10^5^ cells were seeded in a 6-well plate and incubated for 24 h. Then, the cells were exposed to analogs for 48 h. After exposure, the cells were washed to remove the exposure solution. Trypsinized cells were washed twice with PBS and fixed with 70% ice-cold ethanol for 12 h at 4 °C. Then, the cells were washed thrice with PBS and suspended in a 1mg/mL propidium iodide (PI) solution containing 0.1 mg/mL RNase and incubated for 1 h in the dark. The stained cells were subjected to a BD FACS Calibur™ Flow Cytometer (Becton Dickinson, Franklin Lakes, NJ, USA), with the untreated stained cells acting as the controls and the untreated unstained cells acting as the blanks. The data were analyzed using BD CellQuest Pro™ Software (Version 6.1).

### 3.10. Apoptotic Gene Expression after Treatment with BP and EP

The expression of apoptotic genes was evaluated to understand the mechanism at the molecular level [57]. Briefly, HEp-2 cells were seeded at 1 × 10^5^ in a 6-well plate and incubated for 24 h and treated with an analog for 48 h. The total RNA was isolated with TRIzol reagent (BioLit, SRL, Chennai, India) following our laboratory standardized protocol. qPCR for *BCL-2* (B-cell lymphoma-2), *BAX* (Bcl-2 Associated X-protein), *CASP3* (Caspase-3), and *CASP9* (Caspase-9) was carried out using KAPA SYBR FAST one-step qRT-PCR master mix kit in the Light cycler 96 (Roche Diagnostics GmbH, Germany). The primer sequence used for the assay is listed in Table 2. The relative fold change in the expression was analyzed using the 2^−ΔΔCt^ method after normalizing with *GAPDH*, the internal reference gene [58].

### 3.11. Molecular Docking and Dynamic Simulation

The 3D crystal structure of human Cytochrome P450 1A2 (CYP1A2, PDB ID: 2HI4), Dual specificity tyrosine-phosphorylation-regulated kinase 1A (DYRK1A, PDB ID: 7A4O), Dual specificity tyrosine-phosphorylation-regulated kinase 1B (DYRK1B, PDB ID: 7A4O), and Dual specificity protein kinase (CLK1, PDB ID: 1Z57) proteins were retrieved by a research collaborator from the structural bioinformatics protein data bank (RCSB-PDB) (https://www.rcsb.org/ accessed on 28 July 2022). The molecular interaction of BP with the predicted target proteins was carried out using AutoDock Tools 4.0 [60]. Input files such as pdbqt of both the BP and target proteins, grid parameter, and docking parameter were prepared as previously described using AutoDock [61]. BP with a minimum binding energy (ΔG) towards the target proteins during the post-docking analysis was considered for a further molecular simulation analysis [62]. Molecular dynamic simulations of 100,000 picoseconds (ps) were performed to examine the flexibility and stability of the analog BP with CYP1A2 and DYRK1A enzymes at 300 K using the Groningen Machine for Chemical Simulations (GROMACS 5.1.2) package [63]. System preparation, system equilibration, and post-equilibration procedures were performed as stated in the Methods in Supplementary Materials. Analyses of the root-mean-square deviation (RMSD), root-mean-square fluctuation (RMSF), and Radius of gyration (Rg) between the protein and BP in each frame were accomplished using the GROMACS command utilities. 

### 3.12. Statistical Analysis

The data provided in this study are the mean ± standard deviation (SD) of three individual experiments. The obtained data were subjected to ANOVA, followed by post hoc Dunnett’s multiple comparisons to compare the statistical significance level. We used GraphPad Prism software (Ver. 5.03) for the statistical analysis. A threshold was set at *p* < 0.05, which was statistically significant.

## 4. Conclusions

Benzo[b]thiophene analogs with/without a hydroxyl group were evaluated for the ability to inhibit the proliferation of HEp2, AGS, MCF7, and MG63. The antiproliferative activity was compared with those of the respective hydroxyl-masked negative analogs. Analogs 1(3-hydroxybenzo[b]thiophen-2-yl) ethanone (BP) and 1-(3-hydroxybenzo[b]thiophen-2-yl) propan-1-one hydrate (EP) showed potential cytotoxic activity against laryngeal cancer HEp2 cells. Apoptosis induction with an increase in the *BAX/BCL-2* ratio was anticipated to be the mode of inhibitory action. BP and EP analogs could arrest the cells at the Sub-G1 phase, which also indicates apoptosis. Chemotherapeutic drugs are often associated with an increase in oxidative stress, leading to DNA damage, with most of them having a negative effect on the fast-dividing healthy cells. The incorporation of a potent antioxidant in anticancer therapy may reduce the negative effect caused by the pro-oxidant-inducing drugs. The antiproliferative effect of BP and EP is low as a standalone drug, therefore it is recommended that they should be combined with other drugs for effective chemotherapy, since BP and EP possess an antioxidant effect that might ease the effect of pro-oxidant drugs on healthy cells. Combinational therapy using hydroxyl-containing benzo[b]thiophene analogs with the anticancer drug would be a promising approach in the future since BP could possibly inhibit CYP1A2, which allows the anticancer agents to have a longer working duration in the cells to exert their mechanism, thereby increasing the efficiency of the therapy. The CYP1A2 inhibition-mediated beneficial action of BP has to be studied thoroughly before combinational therapy is offered to circumvent the therapeutic failure. 

## Figures and Tables

**Table 1:** Growth inhibitory concentration (IC_20_ and IC_50_) values of benzo[b]thiophene analogs against various cancer cell lines as per MTT assay.

Cell Lines	BP (µM)		BN (µM)		EP (µM)		EN (µM)	
	IC_20_	IC_50_	IC_20_	IC_50_	IC_20_	IC_50_	IC_20_	IC_50_
**HEp2** Laryngeal carcinoma	6.01 ± 0.98	**27.02 ± 1.23**	35.28 ± 2.4 ^¶^	195.9 ± 3.11 ^¶^	3.89 ± 1.06	35.26 ± 2.15 *	36.05 ± 2.56 ^§^	364.7 ± 4.31 ^§^
**AGS** Gastric adenocarcinoma	16.5 ± 1.23	74.44 ± 4.07	91.7 ± 3.36 ^¶^	279.1 ± 7.19 ^¶^	11.91 ± 1.64	67.64 ± 3.08 *	131.2 ± 4.54 ^§^	292.2 ± 8.23 ^§^
**MG63** Osteosarcoma	27.48 ± 2.05	129.4 ± 2.06	84.07 ± 3.57 ^¶^	279.1 ± 8.15 ^¶^	12.17 ± 1.24	51.31 ± 3.06 **	30.97 ± 2.8 ^§^	292.2 ± 4.2 ^§^
**MCF7** Breast adenocarcinoma	11.41 ± 1.36	81.92 ± 2.09	47.29 ± 2.74 ^¶^	154.6 ± 7.15 ^¶^	9.9 ± 2.14	54.75 ± 3.11 *	52.14 ± 3.26 ^§^	239.9 ± 5.12 ^§^
**Vero** Kidney epithelial cells (non-tumorogenic)	-	138.57 ± 7.9	-	202.5 ± 5.68	-	103.58 ± 8.57	^-^	324.2 ± 6.85
**Selectivity Index** (**SI**) for HEp2 cells		**5.13**		>1		**2.94** **		>1

IC_20_ and IC_50_ values are presented as mean ± SD, * represents *p* < 0.05 and ** represents *p* < 0.01 (* or ** denotes significance between BP and EP treatments), ¶ represents *p* < 0.01 (¶ denotes significance between the BP and BN treatments), and § represents *p* < 0.01 (§ denotes significance between EP and EN treatment).

**Table 2:** List of apoptosis-related gene primers used in the study.

Gene	Forward Primer (5′–3′)	Reverse Primer (5′–3′)	Reference
*GAPDH*	GTCTCCTCTGACTTCAACAGCG	ACCACCCTGTTGCTGTAGCCAA	[59]
*BAX*	TCAGGATGCGTCCACCAAGAAG	TCAGGATGCGTCCACCAAGAAG	[59]
*BCL-2*	GTGGATGACTGAGTACCT	CCAGGAGAAATCAAACAGAG	[59]
*CASPASE 3*	ACATGGAAGCGAATCAATGGACTC	AAGGACTCAAATTCTGTTGCCACC	[59]
*CASPASE 9*	GCTCTTCCTTTGTTCATC	CTCTTCCTCCACTGTTCA	[59]

## Data Availability

Data will be made available on request.

## Supplementary Materials

The following supporting information can be downloaded at: https://www.mdpi.com/article/10.3390/molecules28041856/s1. Figure S1: ^1^H NMR spectrum of BP in CDCl_3_; Figure S2: ^13^C NMR spectrum of BP in CDCl_3_; Figure S3: ^1^H NMR spectrum of BN in CDCl_3_; Figure S4: ^13^C NMR spectrum of BN in CDCl_3_; Figure S5: ^1^H NMR spectrum of EP in CDCl_3_; Figure S6: ^13^C NMR spectrum of EP in CDCl_3_; Figure S7: ^1^H NMR spectrum of EN in CDCl_3_; Figure S8: ^13^C NMR spectrum of EN in CDCl_3_; Figure S9: HRMS spectrum of BP; Figure S10: HRMS spectrum of BN; Figure S11: HRMS spectrum of EP; Figure S12: HRMS spectrum of EN; Figure S13: Molecular simulation dynamics of CYP1A2 (Grey) and DYRK1A (Red) protein and ligand BP. (A) Minimum distance and (B) total interaction around 3.5 Å of CYP1A2 and DYRK1A protein relative to the starting complexes during 10,000 ps MD test; Figure S14: Energetic components by MD simulation show a better interaction of BP with CYP1A2 than with DYRK1A.

## Sample Availability

Samples of the compounds BP, BN, EP, and EN are available from the corresponding authors, Pushparathinam Gopinath and Jesu Arockiaraj, upon reasonable request.
