# Supplementary material for: Functionalized Sulfur-Containing Heterocyclic Analogs Induce Sub-G1 Arrest and Apoptotic Cell Death of Laryngeal Carcinoma In Vitro
Source: Molecules. 2023 Feb 15;28(4):1856. doi: 10.3390/molecules28041856 (PMC9963856; doi:10.3390/molecules28041856)
Supplement: Supplementary file 1 [file molecules-28-01856-s001.zip › molecules-2079010-supplementary.pdf]

Supplementary Materials

Functionalized Sulfur-Containing Heterocyclic Analogs Induce Sub-G1 Arrest and Apoptotic Cell Death of Laryngeal Carcinoma In Vitro

B. Haridevamuthu [1], Tamilvelan Manjunathan [2], Carlton Ranjith Wilson Alphonse [3], Rajendran Saravana Kumar [4], Sundaram Thanigaivel [1], Somasundaram Chandra Kishore [5], Vickram Sundaram [6], Pushparathinam Gopinath [2,*], Jesu Arockiaraj [1,*] and Stefano Bellucci [7,*]

1 Department of Biotechnology, College of Science and Humanities, SRM Institute of Science and Technology, Kattankulathur, Chennai 603203, Tamil Nadu, India
2 Department of Chemistry, College of Engineering and Technology, SRM Institute of Science and Technology, Kattankulathur, Chennai 603203, Tamil Nadu, India
3 Molecular and Nanomedicine Research Unit, Centre for Nanoscience and Nanotechnology, Sathyabama Institute of Science and Technology, Chennai 600119, Tamil Nadu, India
4 Chemistry Division, School of Advanced Sciences, VIT University, Chennai Campus, Chennai 600127, Tamil Nadu, India
5 Department of Biomedical Engineering, Saveetha School of Engineering, Saveetha Institute of Medical and Technical Sciences, Chennai 602105, Tamil Nadu, India
6 Department of Biotechnology, Saveetha School of Engineering, Saveetha Institute of Medical and Technical Sciences, Chennai 602105, Tamil Nadu, India
7 INFN—Laboratori Nazionali di Frascati, 00044 Frascati, Italy
* Correspondence: gopinaat03@gmail.com (P.G.); jesuaroa@srmist.edu.in (J.A.); stefano.bellucci@lnf.infn.it (S.B.)

Table of Content Pages

1. Synthesis procedure and analytical data 1-5
2. Figure S1 and S2: ^{1}H and ^{13}C NMR of BP 6
3. Figure S3 and S4: ^{1}H and ^{13}C NMR of BN 7
4. Figure S5 and S6: ^{1}H and ^{13}C NMR of EP 8
5. Figure S7 and S8: ^{1}H and ^{13}C NMR of EN 9
6. Figure S9-Fig S12 HRMS data of BP, BN, EP, and EN 10
7. Figure S13-Fig S14 MD simulation studies 11-12

General experimental information

All the reactions were performed with the commercially available starting materials without any further purifications. Proton nuclear magnetic resonance (^{1}H NMR) spectra were recorded using a BrukerBBFO (500 MHz) spectrometer (Fällanden, Switzerland). Chemical shifts were recorded in parts per million (ppm, δ) relative to chloroform (δ = 7.26, singlet). ^{1}H NMR splitting patterns are designated as singlet (s), doublet (d), triplet (t), quartet (q), doublet of doublets (dd), multiplet (m), broad singlet (bs) ones, etc. Carbon nuclear magnetic resonance (^{13}C NMR) spectra were recorded using a BrukerBBFO (126 MHz) spectrometer. ^{13}C NMR data are reported with the solvent peak ($CDCl_3$ =77.16) as the internal standard. High-resolution mass spectral analysis (HRMS) was performed using Bruker Impact HD mass spectrometer. Analytical thin-layer chromatography (TLC) was carried out on a Merck 60 F254 pre-coated silica gel plate (0.2 mm thickness) (Ontario, Canada). Visualization was performed using a UV lamp. Melting points were recorded with Deep vision melting point 935LED (Chennai, India). The instrument was calibrated with benzoic acid before the measurement.

Synthesis of 1-(3-hydroxybenzo[b]thiophen-2-yl) ethanone (BP)

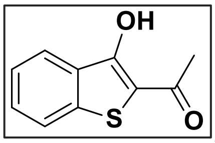Sodium hydride (1.2 g, 4 equiv.) was added to a round bottom flask containing 10 mL of dry tetrahydrofuran (THF), and to this solution, acetylacetone (1.9 mL, 2.2 equiv.) was added dropwise at 0 °C, and the mixture was stirred for 30 minutes. Following that, freshly prepared dithiodibenzoyl chloride (2.8 g, 1 equiv.) was slowly added to dry THF (15 mL), the mixture was allowed to warm to room temperature, and stirring was continued for 4 days. After extracting the reaction mixture with ethyl acetate (80 mL), the organic layer was washed with water (75 mL) and dried over anhydrous Na_2SO_4. It was filtered, and the solvent was evaporated under reduced pressure, yielding a residue that was chromatographed on silica gel in a gradient mode with EtOAc/hexanes to yield the compound BP (650 mg; yield 84%). 1 H NMR (500 MHz, $CDCl_3$) δ 12.27 (bs, 1H), 7.98 (td, J = 8.05, 0.9 Hz, 1H), 7.73 (td, 8.2, 0.7 Hz, 1H), 7.54 (ddd, J = 7.65, 1.25, 0.9 Hz, 1H), 7.41 (ddd, J = 7.65, 0.9, 0.9 Hz, 1H), 2.50 (s, 3H). 13C NMR (126 MHz, $CDCl_3$) δ 196.93, 161.74, 139.17, 130.54, 129.95, 124.90, 123.94, 123.41, 111.61, 28.35. HRMS: m/z Calcd. for ($C_{10}H_8O_2S$): 192.0318; Found: [M+H] 193.0321.

Synthesis of 2-acetylbenzo[b]thiophene-3-yl acetate (BN)

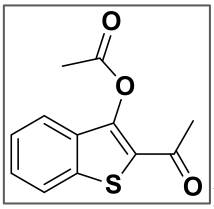In a 25 mL round bottom flask, we dissolved BP (1 equiv.) in 3 mL dichloromethane (DCM) and stirred for 20 minutes at 0 °C with triethylamine (2 equiv.). To this stirred solution, we added acetyl chloride (1.5 equiv.) and stirred until the starting material disappeared (3-4h). Twenty-five mL of water was used to wash the reaction mixture, and it was dried over anhydrous Na_2SO_4. It was filtered, and the filtered solvent was concentrated under a reduced pressure to yield a residue that was chromatographed on silica gel in a gradient mode using EtOAc/hexanes to obtain the compound BN. 1 H NMR (500 MHz, $CDCl_3$) δ 7.81 (d, J = 8.2 Hz, 1H), 7.64 (td, J = 8.2, 0.85 Hz, 1H), 7.51 (ddd, J = 7.75, 1.2, 1.2 Hz, 1H), 7.43 (ddd, J = 7.6, 0.9, 0.9 Hz, 1H),2.59 (s, 3H), 2.51 (s, 3H), 13C NMR (126 MHz, CDCl3) δ 190.66, 168.27, 143.80, 138.17, 132.94, 129.32, 128.48, 125.36, 123.42, 122.81, 29.50, 20.95. HRMS: m/z Calcd. for ($C_{12}H_{10}O_3S$): 234.0423; Found: [M+H] 235.0418.

Synthesis of 1-(3-hydroxybenzo[b]thiophen-2-yl) propan-1-one hydrate (EP)

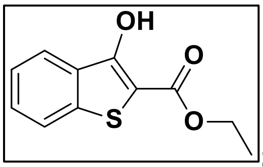Sodium hydride (1.2 g, 4 equiv.) was added to a round bottom flask containing 10 mL of dry tetrahydrofuran (THF), and to this solution, ethyl acetoacetate (2.6 mL, 2.2 equiv.) was added dropwise at 0 °C, and the mixture was stirred for 30 minutes. Following that, freshly prepared dithiodibenzoyl chloride (2.8 g, 1 equiv.) was slowly added to dry THF (15 mL), and the mixture was allowed to warm to room temperature, and stirring was continued for 4 days. After extracting the reaction mixture with ethyl acetate (80 mL), the organic layer was washed with water (75 mL) and dried over anhydrous Na_2SO_4. It was filtered, and the solvent was evaporated under a reduced pressure, yielding a residue that was chromatographed on silica gel in a gradient mode with EtOAc/hexanes to yield the compound EP (850 mg; yield 94 %).^{1}H NMR (500 MHz, $CDCl_3$) δ 10.20 (bs, 1H), 7.94(td, J = 8.0, 0.9 Hz, 1H), 7.73 (d, *J* = 8.2 Hz, 1H), 7.50 (ddd, *J* = 8.2, 1.1,

1.2 Hz, 1H), 7.40(ddd, J= 7.1, 0.9,0.9 Hz, 1H), 4.42 (q, *J* = 7.1 Hz, 2H), 1.42 (t, *J* = 7.1 Hz, 3H), ^{13}C NMR (126 MHz, $CDCl_3$) δ 167.47, 159.59, 138.93, 130.57, 128.91, 124.52, 123.25, 123.06, 102.12, 61.52, 14.46. HRMS: m/z Calcd. for ($C_{11}H_{10}O_3S$): 223.0435; Found: [M+H] 223.0428.

Synthesis of 1-(3-methoxybenzo[b]thiophene-2-yl) propan-1-one (EN)

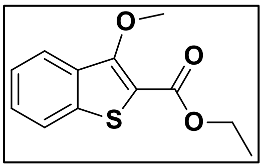

In a 25 mL round bottom flask, we dissolved EP (100 mg 1 equiv.) in 3 mL acetone and K_2CO_3 (103 mg 2 equiv.) and stirred for 20 minutes at room temperature. To this stirred solution, we added methyl iodide (55 μL 2 equiv.) and stirred until the starting material disappeared (2 h). Then, the reaction mixture was filtered, and the filtered solvent was concentrated under reduced pressure, then the crude was chromatographed on silica gel in a gradient mode using EtOAc/hexanes to obtain the compound EN. ^{1}H NMR (500 MHz, $CDCl_3$) δ 7.88 (d, *J* = 8.0 Hz, 1H), 7.74 (d, *J* = 8.1 Hz, 1H), 7.47 (t, *J* = 7.5 Hz, 1H), 7.40 (t, *J* = 7.5 Hz, 1H), 4.39 (q, *J* = 7.1 Hz, 2H), 4.16 (s, 3H), 1.41 (t, *J* = 7.1 Hz, 3H), ^{13}C NMR (126 MHz, $CDCl_3$) δ 162.02, 156.86, 138.18, 133.93, 128.02, 124.62, 123.96, 123.06, 115.64, 62.80, 61.38, 14.45. HRMS: m/z Calcd. for ($C_{12}H_{12}O_3S$): 237.0580; Found: [M+H] 237.0588.

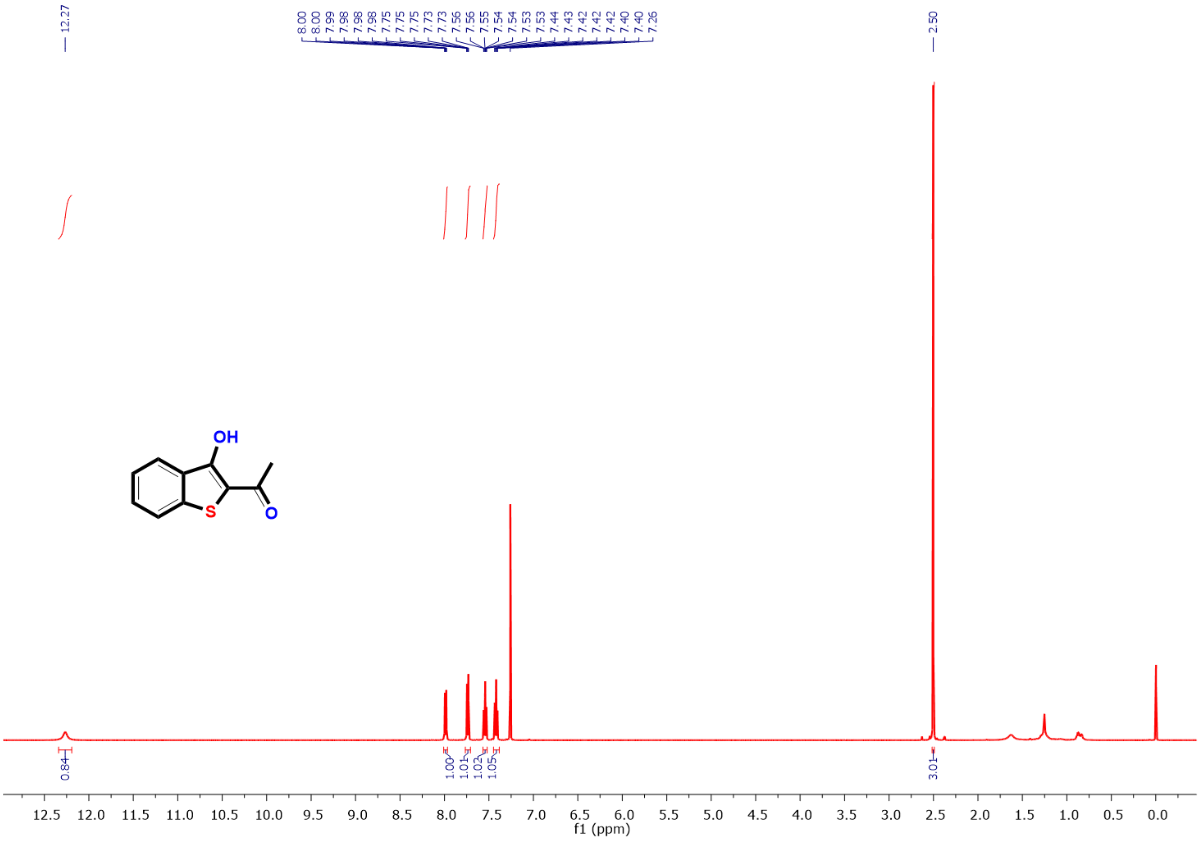

Figure S1. ^{1}H NMR spectrum of BP in $CDCl_3$.

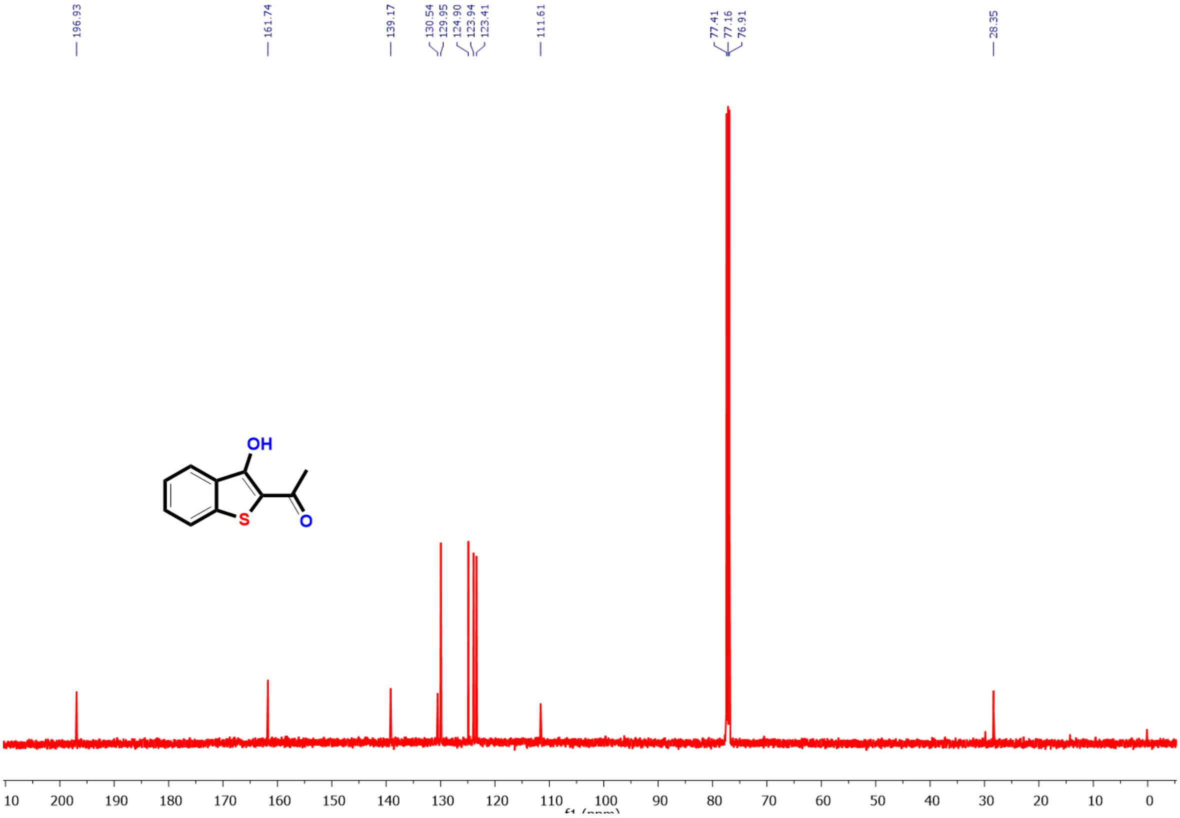

Figure S2. ^{13}C NMR spectrum of BP in $CDCl_3$.

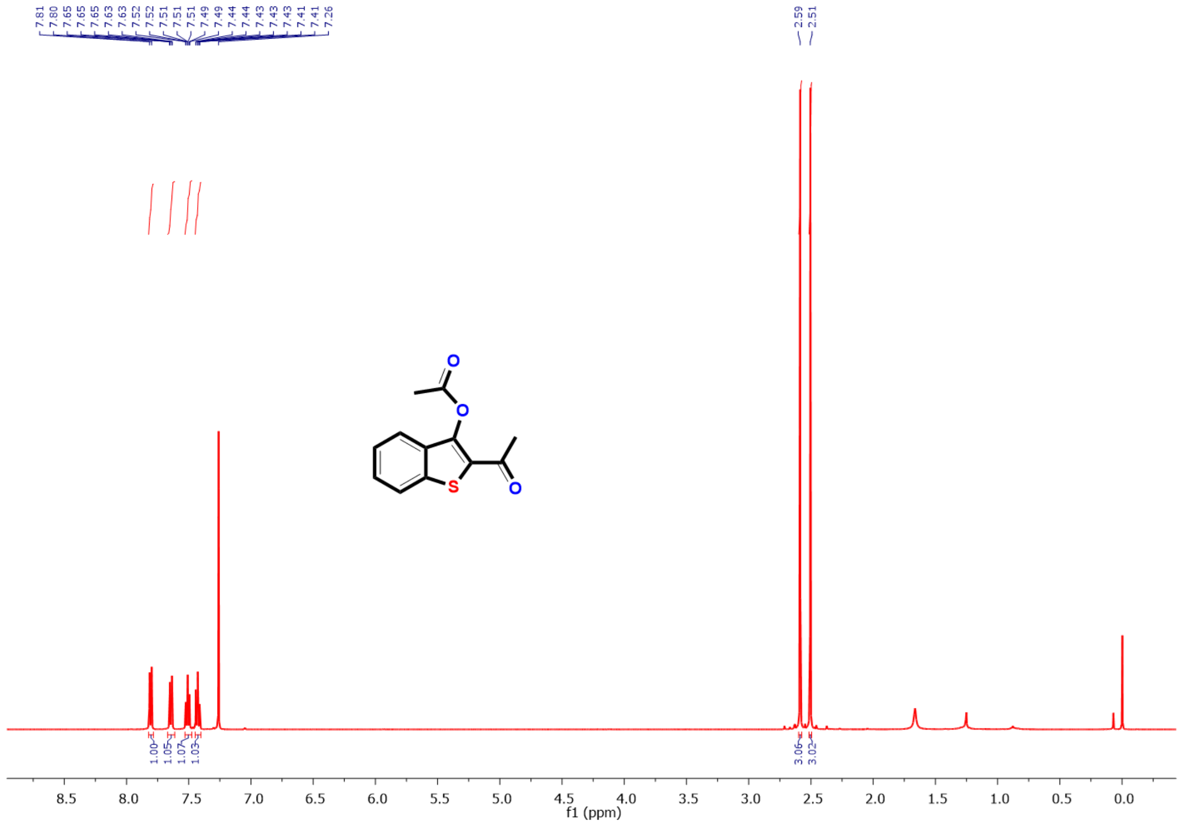

Figure S3. ^{1}H NMR spectrum of BN in $CDCl_3$.

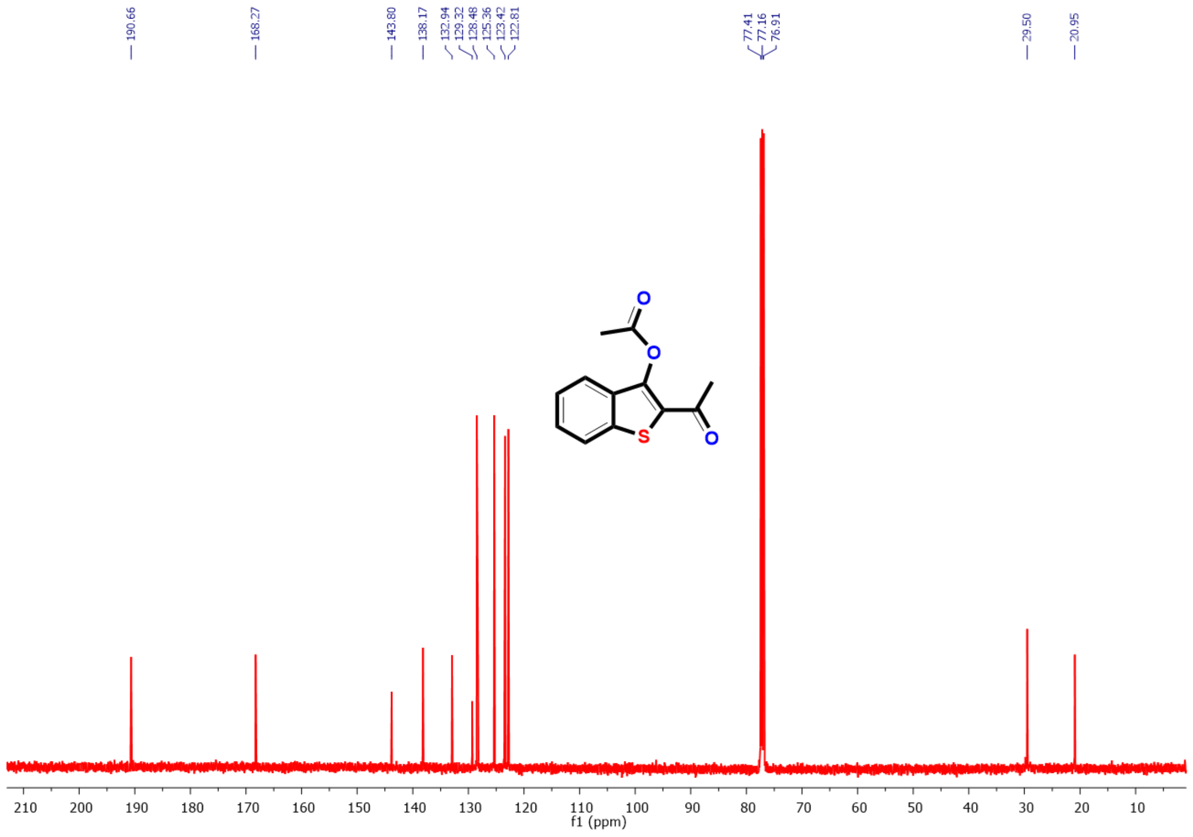

Figure S4. ^{13}C NMR spectrum of BN in $CDCl_3$.

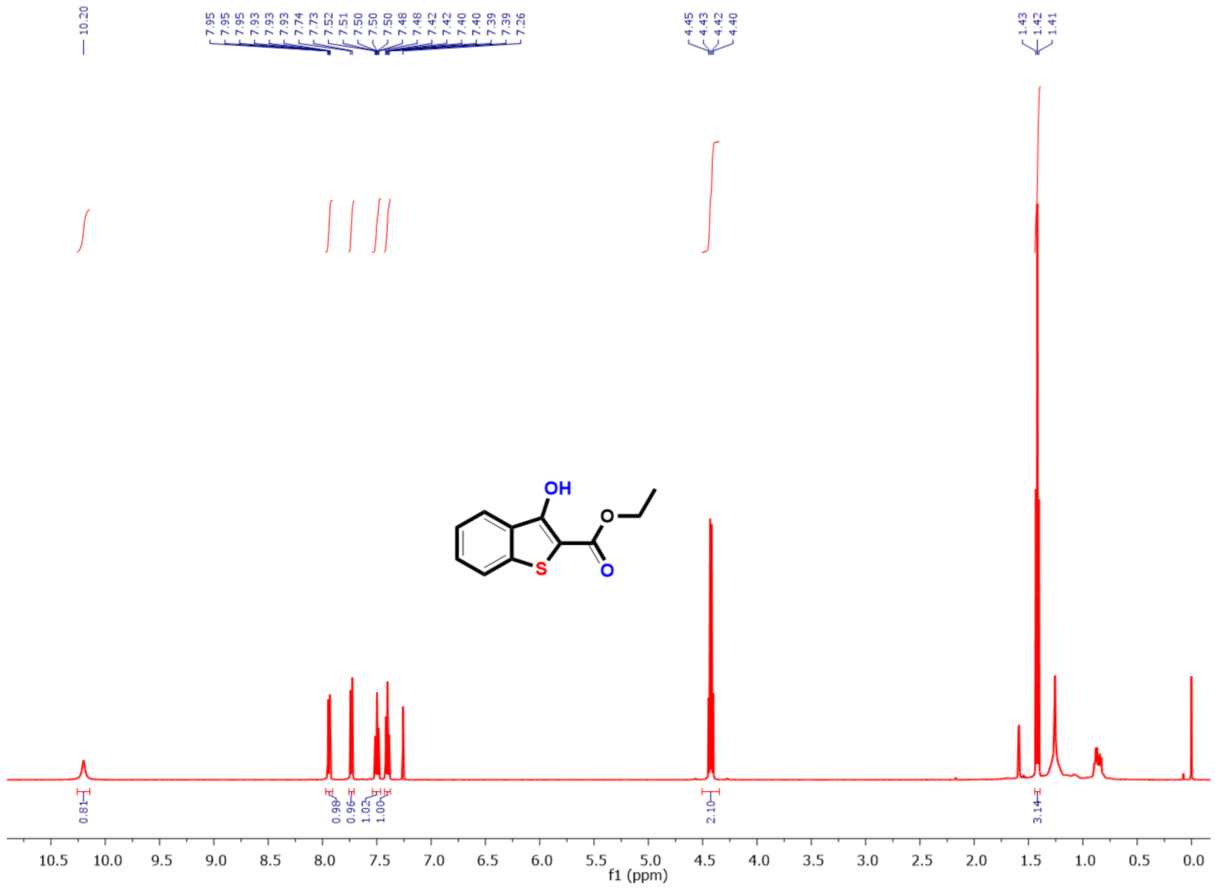

Figure S5. ^{1}H NMR spectrum of EP in $CDCl_3$.

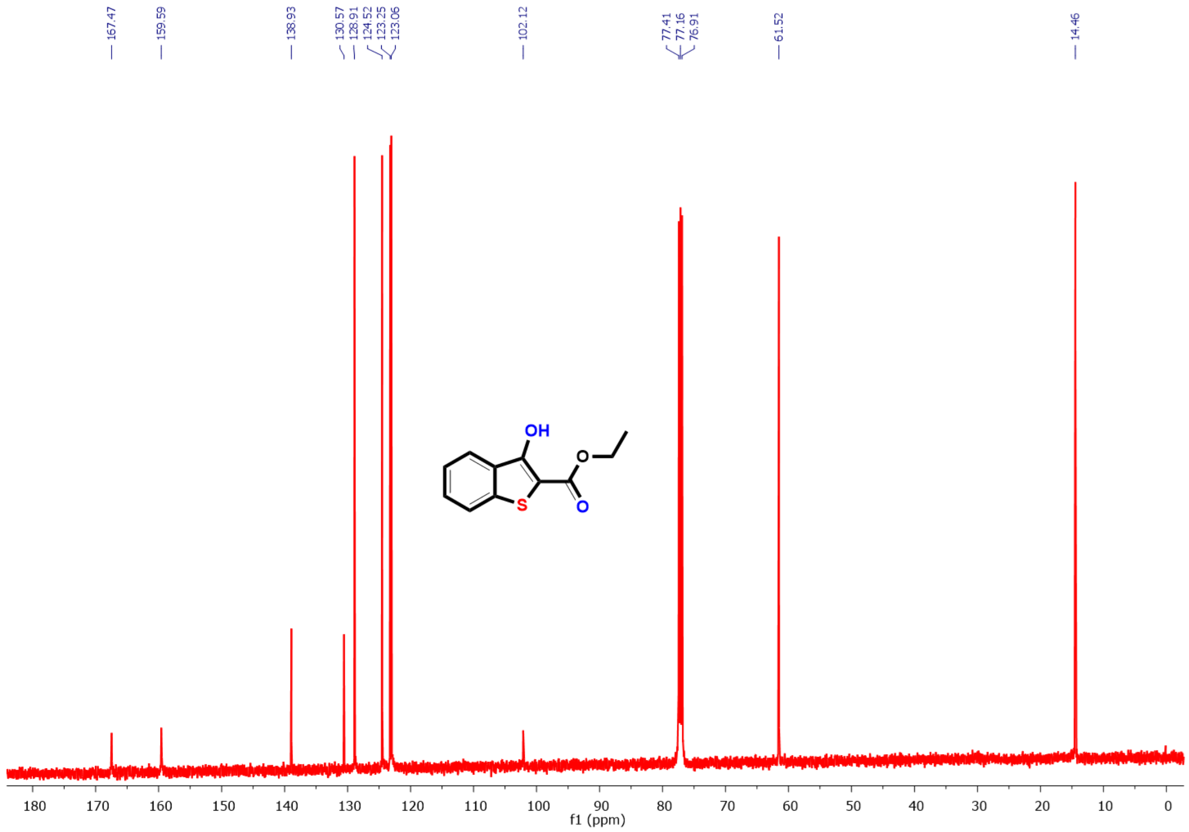

Figure S6. ^{13}C NMR spectrum of EP in $CDCl_3$.

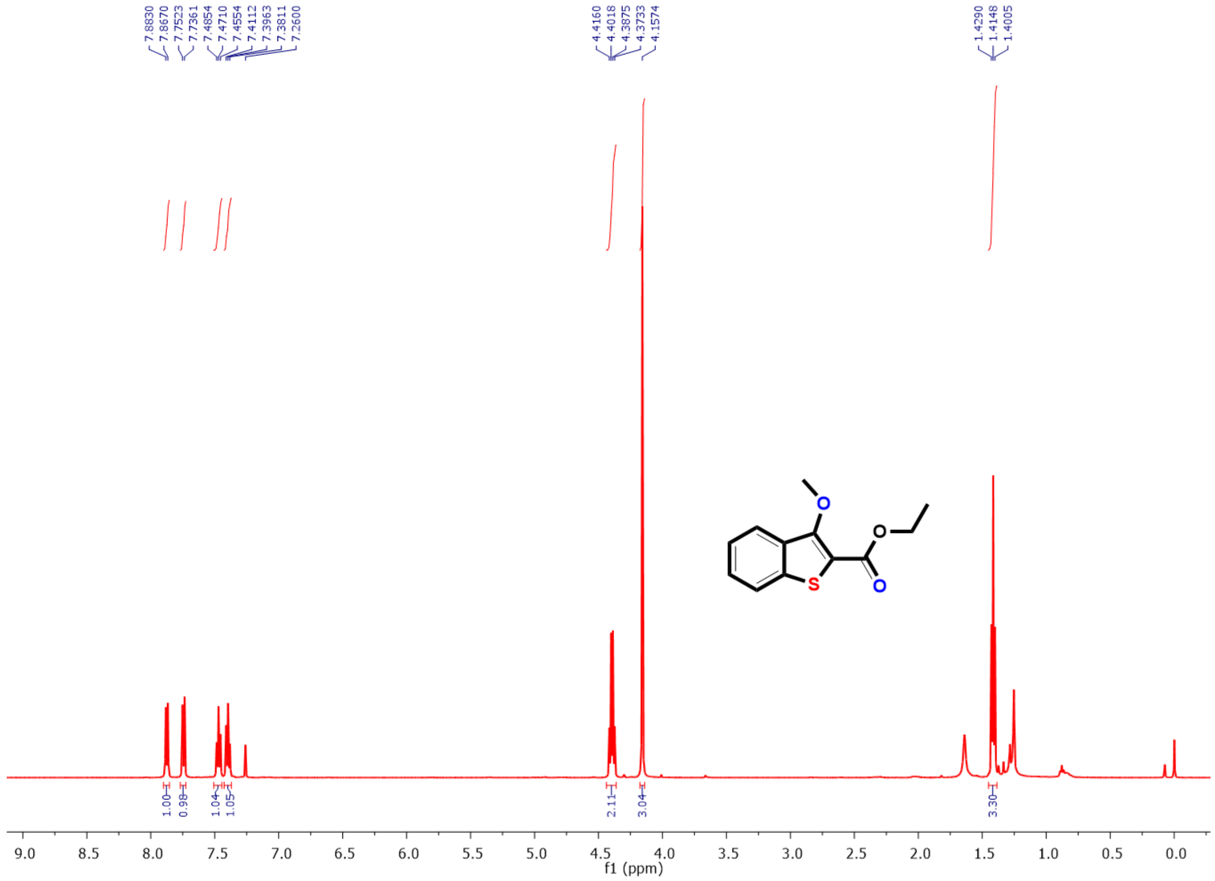

Figure S7. ^{1}H NMR spectrum of EN in $CDCl_3$.

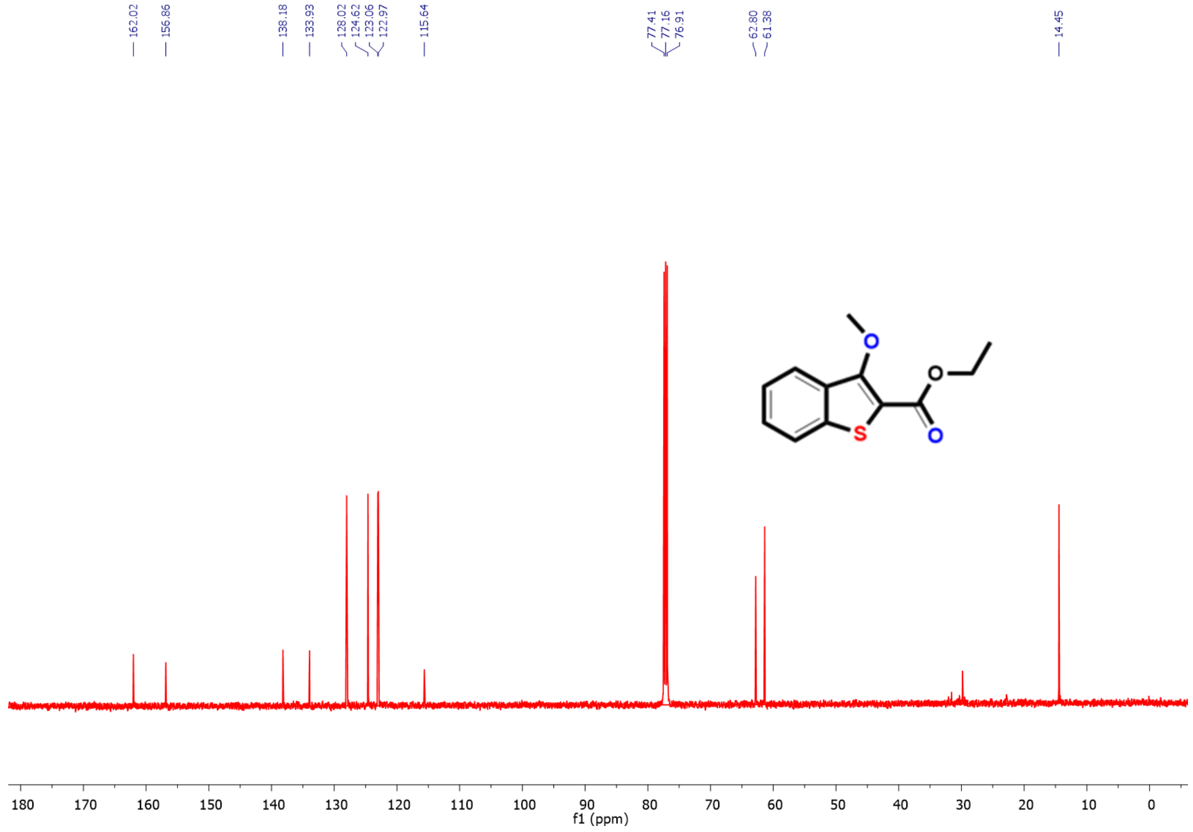

Figure S8. ^{13}C NMR spectrum of EN in $CDCl_3$.

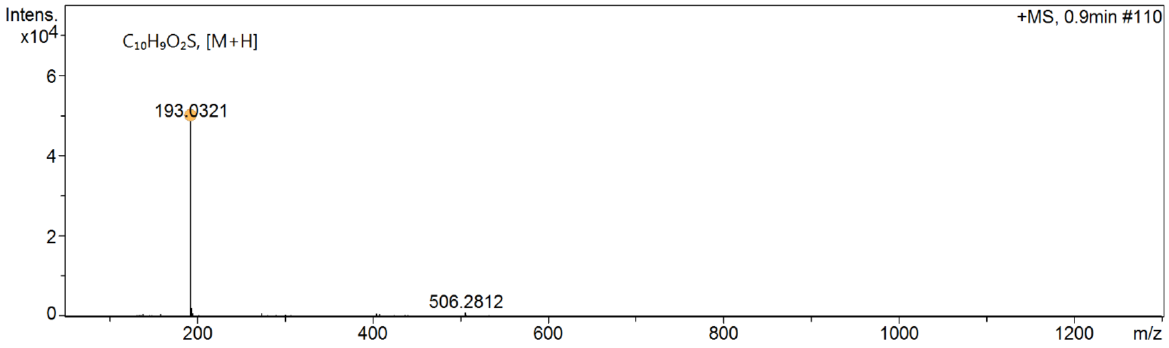

Figure S9. HRMS spectrum of BP.

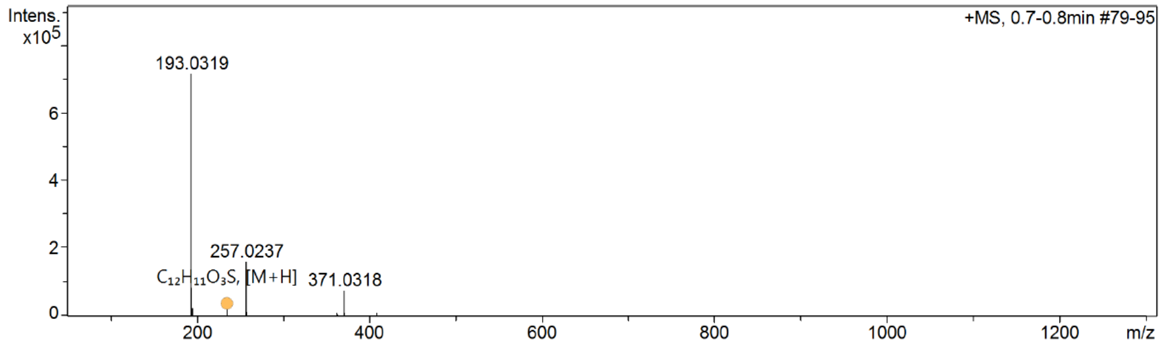

Figure S10. HRMS spectrum of BN.

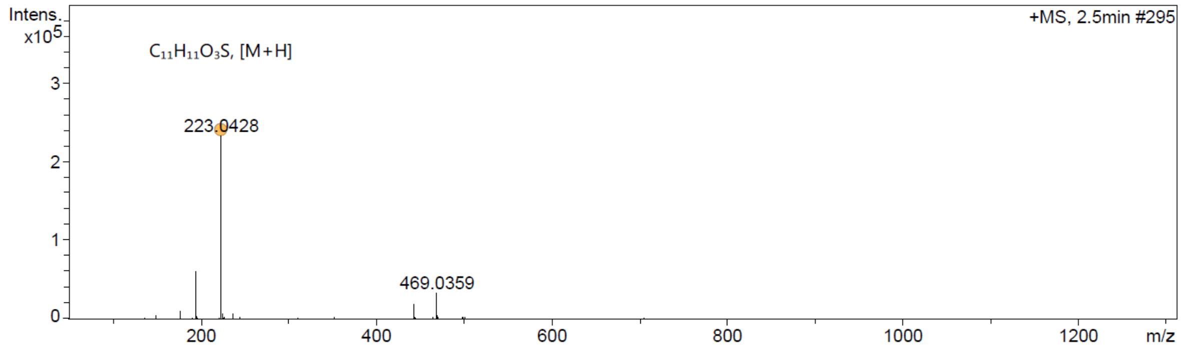

Figure S11. HRMS spectrum of EP.

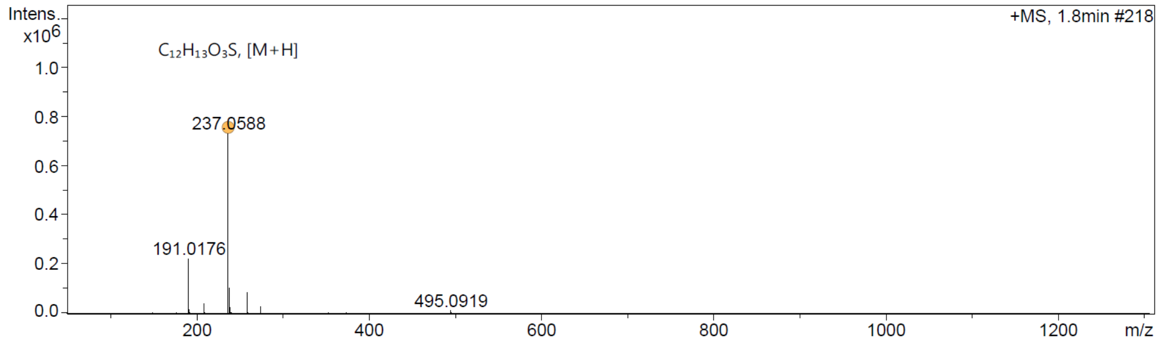

Figure S12. HRMS spectrum of EN.

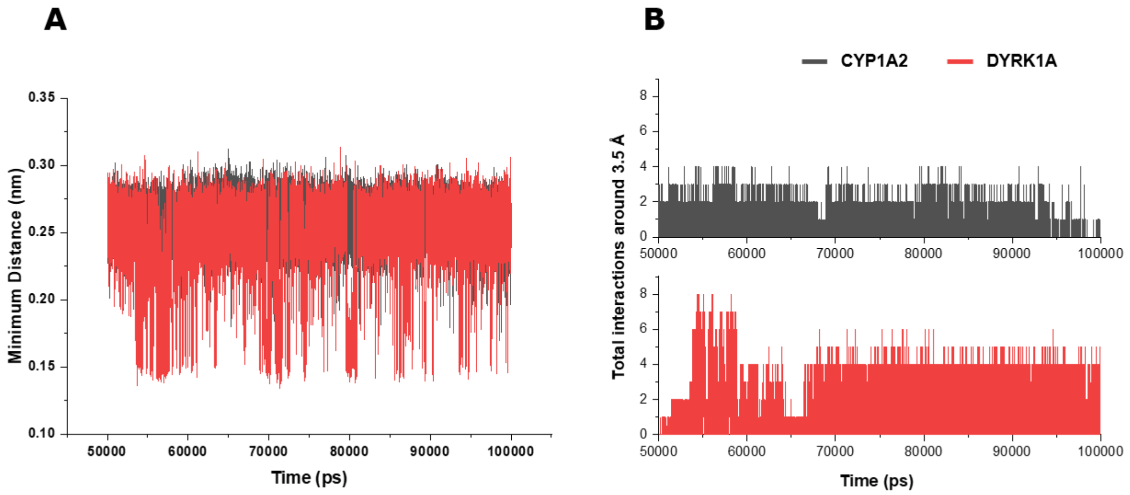

Figure S13. Molecular simulation dynamics of CYP1A2 (Grey) and DYRK1A (Red) protein and ligand BP. **A.** Minimum distance and **B.** Total interaction around 3.5 Å of CYP1A2 and DYRK1A protein relative to the starting complexes during 10000 ps MD test.

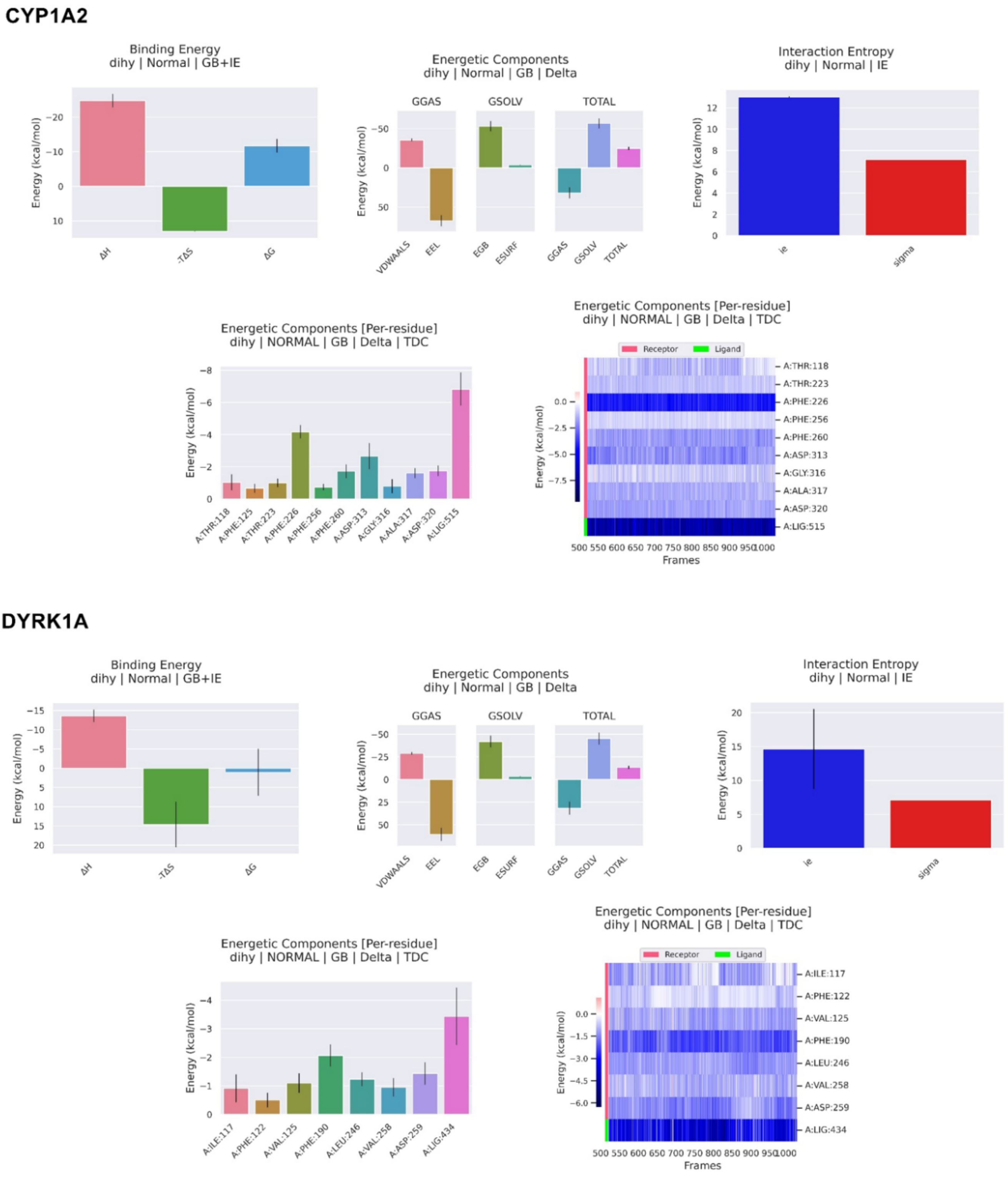

Figure S14. Energetic components by MD simulation show a better interaction of BP with CYP1A2 than with DYRK1A.
